# Intensity and exposure proximity as determinants of differential stress-related health outcomes

**DOI:** 10.1038/s41380-026-03515-5

**Published:** 2026-03-06

**Authors:** Maya Lebow, Ron Rotkopf, Elad Malik, Maya Amitai, Abraham Weizman, Ofra Krieger, Akim Geyshin, Ariel Israel, Eugene Merzon, Eli Magen, Shlomo Vinker, Orit Nuttman-Shwartz, Alon Chen

**Affiliations:** 1 Department of Brain Sciences, Weizmann Institute of Science, Rehovot, Israel; 2 Department of Molecular Neuroscience, Weizmann Institute of Science, Rehovot, Israel; 3 Bioinformatics Unit, Department of Life Sciences Core Facilities, Weizmann Institute of Science, Rehovot, Israel; 4 Leumit Health Services, HMO, Tel Aviv, Israel; 5 Abarbanel Mental Health Center, Bat Yam, Israel; 6 Gray Faculty of Medical & Health Sciences, Tel Aviv University, Tel Aviv, Israel; 7 Felsenstein Medical Research Center, Petach Tikva, Israel; 8 Research Unit, Geha Mental Health Center, Petah Tikva, Israel; 9 The Adelson School of Medicine, Ariel University, Ariel, Israel; 10 Samson Assuta Hospital, Ashdod, Israel; 11 Faculty of Health Sciences, Ben Gurion University of the Negev, Beer Sheva, Israel; 12 School of Social Work, Sapir College, D.H. Hof Ashkelon, Sderot, Israel

**Keywords:** Predictive markers, Neuroscience

## Abstract

Civilian populations under continuous traumatic stress (CTS) face long-term health consequences mediated primarily by glucocorticoid stress pathways. To assess the health impact of exposure to CTS and subsequent acute trauma, we conducted a retrospective observational study of 208,625 adults in southern and central Israel exposed to CTS and aftermath of October 7, 2023 attacks. Using anonymized electronic health records (2005–2024) and a serial cross-sectional design stratified by exposure level, sex, and age, we assessed psychiatric morbidity, medication use, immune disease, mortality, and blood biomarkers. CTS exposure level was defined by residential proximity to the Gaza border as high, moderate, or low (15, 30–60, and 90 seconds to shelter, respectively), and acute trauma effects were assessed by comparing ten months before and after October 7.

Prior to October 7, psychiatric morbidity increased across all exposure groups, with moderately exposed women showing the highest baseline prevalence. Following October 7, morbidity rose in an exposure-dependent manner, among highly exposed women, with predominant antidepressant use compared with higher anxiolytic use among moderately exposed women. Highest vulnerability was observed in ages 46–64.

Sex-specific interactions were observed, including autoimmune disease and elevated MCV among moderately exposed women, and pneumonia with elevated WBC among highly exposed men. GGT was linked to reduced psychiatric risk in men, whereas elevated alkaline phosphatase was associated with increased autoimmune risk in women. Highly exposed men showed 374% higher mortality than women and 88% higher mortality than moderately exposed men. Age, sex, and biomarker-based screening may mitigate CTS-related disease.

## Introduction

Exposure to stressful challenges activates complex neuroendocrine, physiological, and behavioral responses designed to ensure survival. Stressors rapidly activate the autonomic nervous system, stimulating the sympatho-neuronal (SN) and sympatho-adrenomedullary (SAM) branches, which release noradrenaline and adrenaline. In parallel, the hypothalamic–pituitary–adrenocortical (HPA) axis stimulates secretion of corticotropin-releasing factor (CRF), adrenocorticotropic hormone (ACTH), and cortisol [1–3].

Acute elevations of cortisol, noradrenaline, and CRF influence stress-related behaviors through limbic brain circuits [4, 5], cardiovascular changes from sustained sympathetic activation and HPA axis dysregulation [6] and modulate peripheral physiology of immune and metabolic function via proinflammatory signaling [7, 8]. While short-term activation facilitates adaptation, repeated or prolonged dysregulation of these systems contributes to allostatic load, shifting the organism toward long-term pathological change [2].

At the immune level, glucocorticoids alter leukocyte trafficking and survival, producing shifts in total white blood cell (WBC) counts and lymphocyte subsets [9]. Chronic stress shifts adaptive immunity, from Th1 to Th2 dominance, enhancing pro-inflammatory Th17 responses, and dysregulating cytokines such as IL-6 and IL-17 [10]. These changes contribute to immune suppression, increasing infection risk and altering hematopoiesis, suppressing erythropoietin and bone marrow function, thereby shifting toward autoimmunity [11]. The liver is particularly sensitive to cortisol and catecholamines which influence hepatic glucose and lipid metabolism, and activation of pro-inflammatory pathways [8, 12]. Additionally, extreme chronic or perceived stress has been linked to telomere shortening, a marker of accelerated aging [13, 14].

Factors influencing the activation and resolution of these varied downstream stress- induced responses include the stressor’s intensity, proximity, predictability, and exposure duration. In preclinical models, proximity to a threat correlates with elevated corticosterone [15]. Repeated exposure to stressors of the same intensity may lead to habituation of behavioral and neuroendocrine responses [16], but unpredictable stressors sustain high corticosterone levels days after exposure, while intensity heightens corticosterone responses [17].

Human studies on 9/11 linked stressors proximity to amygdala hyperactivity and reduction in limbic gray matter [18, 19]. Prior stress exposure can also lead to a hyperactive stress response [20], lowering long-term cortisol levels, leading to PTSD and related immune, metabolic, and cardiovascular dysfunction [21–23]. Following 9/11, physical ailments, including cardiovascular and endocrine conditions, increased significantly [24]. Veterans from Iraq and Afghanistan had a higher risk for autoimmune disorders [25] and a Swedish study showed greater health risks for civilians with early-life stress exposure [26]. Similarly, in the Gaza border, a high-exposure conflict zone, residents showed elevated fasting glucose levels [27], indicating metabolic dysregulation, during escalation periods.

The availability of digital health records spanning 18 years prior to the October 7th, 2023, attack (October 7th) and 10 months following this high-intensity event offers a unique opportunity for an epidemiological study of health trajectories under continuous traumatic stress, CTS and after the acute events of October 7^th^. Hence, our aims were to examine stress-induced psychiatric morbidity, medication use, immune dysregulation, physiological alterations reflected in blood biomarkers, and mortality in relation to (1) CTS exposure levels (2) changes pre and post October 7 reflecting the impact of acute trauma superimposed on CTS; and (3) sex- and age-specific interactions To address these aims, we stratified the population by exposure level (high, moderate, or low), age, and sex. This stratified design allowed isolation of chronic and acute stress effects and their synergistic impact, mirroring controlled preclinical stress paradigms, providing a translational framework for dissection of contributing factors in a civilian population. Stratification enabled robust, subgroup-specific assessment of psychiatric, immune, physiological, and mortality rates across acute and long-term outcomes.

### Cohort

This study was conducted in accordance with the Declaration of Helsinki and all relevant guidelines and regulations. Ethical approval was obtained from the Leumit Health Services Institutional Review Board (LEU-0029-23). The requirement for informed consent was waived by the Institutional Review Board due to the retrospective design of the study and the use of anonymized electronic health records.

This retrospective observational study included anonymized electronic health records from 208,625 active adult members (>18 years) of Leumit Health Services, a national health maintenance organization in Israel serving a predominantly urban population in the southern and central regions (Supplementary Table 1).

We used a serial sectional design [28], stratified [29] by exposure level, sex, and age group (19–45, 46–64, 65+) to assess significant trends related to exposure intensity.

**Fig. 1:**
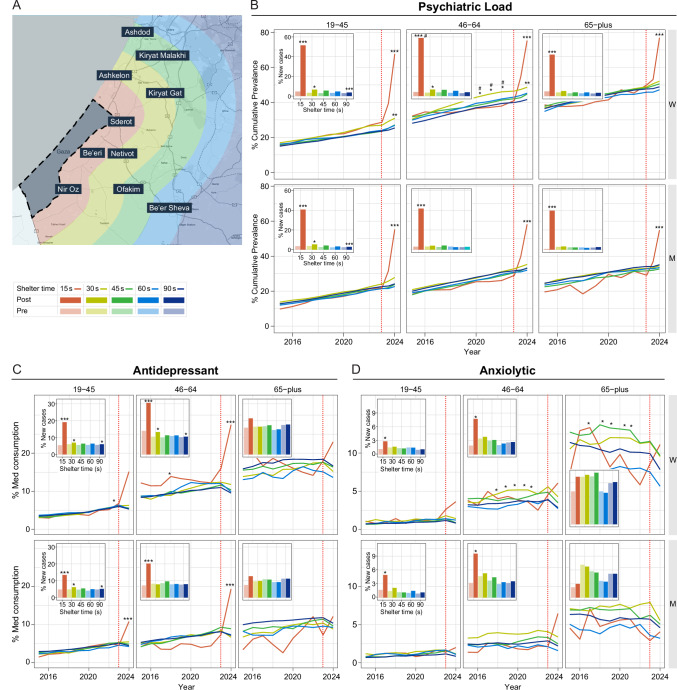
Longitudinal analysis of prevalence rates of psychiatric load and medication usage January 2015-August 2024. **A** Map of distribution of the population by time to shelter as determined by distances from Gaza border (**B**) Pre-October 7th,(to the left of the red dotted line) 30 s continuously exposed women ages 19–45 (top, left panel) and 46–64 (top, center panels) had significantly higher psychiatric load than low exposure areas (2015–2024, p < 0.001, 19–45, 46–64, 30 s vs 90 s,). Women aged 46–64 (top, center panel) had a prevalence of up to 46%, significantly higher than their high exposure counterparts (p < 0.01, 2021–2023 15 s vs 30 s, denoted by “#”). Post-October 7 highly exposed women 46–64 had psychiatric load rates of 66%, and 30 s women had 42%. (p < 0.001, 15 s vs 90 s; p < 0.001, 30 s vs 90 s, p < 0.01,15 s vs 30 s). % new cases pre vs post (center insets) in women and men 19–45 and 46–64 (15 s pre vs 15 s post, 19–45, 36% increase, p < 0.001; 19–45, 30 s pre vs 30 s post, 4% increase, p < 0.01; 90 s pre vs 90 s post, 2% increase, p < 0.001). Antidepressant prescriptions (**C**) were already significantly higher in 15 s pre-October 7th (left of the red dotted line), up to 5% in women 19–45 (2018, 15 s vs 90 s, p < 0.05). Post-Oct 7 (left and center, insets), there was a significant increase of of 14% and 17% in the 19–45 and 46–64 age groups (15 s pre vs post, p < 0.001) and an increase of 13% in men 46–64 men (15 pre vs post, p < 0.001) (center inset). 30 s and 90 s exposed groups had more modest, yet significant, increases within shelter time (30 s pre vs 30 s post, p < 0.05, p < 0.01, 90 s pre vs. post). **D** Use of anxiolytics increased in high exposed 46–64 aged men (15 s pre vs. post, p < 0.05,) and women (15 s pre vs. post, p < 0.001). In 30 s areas (top center, right), women already 3% high rates from 2017–2022 (46–64, 65+ p < 0.05 30 s vs 90 s).

Trauma exposure was defined by residential proximity to the Gaza border and time to reach a shelter zones, as designated by the Israeli Home Front Command (Fig. 1A). Residents within 10 km of the border with 15 seconds to shelter (15 s) were classified as high exposure. Residents 10–70 km from the border had 30–60 seconds to shelter (30 s, 45 s, 60 s), designated as moderate exposure. Residents greater than 70 km from the border 90 seconds (90 s) were classified as low exposure.

Based on historical siren distributions (Supplementary Table 2), the 30 s zone consistently represented the most stable tier of moderate exposure and was used as the reference group, though 45 s and 60 s had stressful, shorter time to shelter than 90 s. While all regions were exposed to warning sirens and rocket fire, the 15 s region was also subject to direct cross-border invasions (Supplementary Table 2) and longest exposure duration. Rocket attacks were recorded in the 15 s zone from 2001, in 30–60 s zones from 2008, and in 90 s zones from 2012. October 7, 2023, marked an extreme acute traumatic event with invasion of border communities, kidnappings, sexual violence, murders, and widespread rocket attacks affecting all shelter zones. All shelter times were exposed to continuous stress at different intensity levels, at different durations and then an acute event of October 7th in varying intensities.

Movements between shelter zones were relatively uncommon.

If a member moved (e.g., from 30 s to 15 s), their data were included in the original zone until the year of relocation, and in the new zone thereafter. Residents of 15 s who were relocated temporarily after October 7^th^, still retained active HMO status and residence in 15 s shelter time.

### Analysis timeline

Psychiatric diagnoses were available from January 2015 to August 2024, when systematic coding was integrated into the database. All other were available from January 2005–August 2024.

### Outcomes

Psychiatric load: Defined as the cumulative prevalence of stress-related psychiatric diagnoses in which any one or more indicate the impact of stress exposure [30] (adjustment disorder, PTSD, anxiety, depression, substance dependence; Supplementary Table 3). To account for underdiagnosis [31], psychiatric load was validated with medication use in four drug classes: antidepressants, antipsychotics, anxiolytics, and hypnotics/sedatives (Supplementary Table 4). Chronic use was defined as more than 2 purchases within a year.

Other outcomes:

- Bacterial pneumonia (Supplementary Table 5)
- Organ-specific autoimmune disorders (Supplementary Table 6).

### Blood laboratory biomarkers

Retrospective results of blood tests were analyzed from complete blood counts and biochemistry panels (Supplementary Table 7). Active members with a test each year were included. Repeated measures across years in individuals were not tracked, preserving statistical power, a serial cross-sectional sampling, with different individuals contributing each year [28]. Values reflect population-level averages. For normal data distribution, log-transformation was performed and variances were similar. The effect size, Cohen’s *d*, was categorized by sizes: small: *d* > 0.2, small to moderate: *d* > 0.4 and moderate *d* > 0.5.

### Statistical analysis

Prevalence estimates were calculated by assessing cumulative diagnoses carried forward from first entry (once diagnosed, an individual remained in the case denominator in subsequent years). New cases were defined as first diagnoses within a given year. Two main comparisons were performed:

1. Impact of shelter time: Cumulative prevalence in one zone compared to others (e.g., 15 s vs. 90 s; 30 s vs. 90 s; 15 s vs. 30 s).
2. Impact of timing (pre vs. post): Within-zone comparisons before vs. after October 7, 2023 (e.g., new cases in the 10 months before vs. 10 months after).
3. Men vs Women: within specific disease aggregates for sex differences independent of exposure [32].

Pairwise comparisons of proportions were conducted using chi-squared tests. Mortality risk was assessed using Cox proportional hazards models with hazard ratios (HR) and 95% confidence intervals (CI). Generalized logistic regression with odd ratio (OR) and 95% CI was used to assess contribution of factors to disease outcome. Laboratory test differences were quantified using Cohen’s d. To address multiple comparisons, we applied the Benjamini–Hochberg procedure to control the false discovery rate at q = 0.05. The correction was performed separately for each shelter-time combination, within each year, sex, age group, and laboratory test, while accounting for all shelter comparisons within the same year, sex, and age group to address dependency among comparisons. All analyses were conducted in R v.4.3.1.

## Results

A cohort of active Leumit HMO members comprised of 208,625 was split by shelter time, as depicted in the map (Fig. 1A), as well as by age groups and sex (Supplementary Table 1). Longitudinal analysis of prevalence rates of psychiatric load demonstrated that prior to October 7, CTS-exposed 30 s women ages 19–45 and 46–64, had significantly higher psychiatric load than 90 s areas (Fig. 1B, top left and center, respectively) during 2015–2024 (p < 0.001, 19–45, 46–64, 30 s vs 90 s). Additionally in all years and in age groups, women had higher prevalence then men (p < 0.001, 2015–2024, 19–45, 46–64, 65+, 15 s women vs men, 30 s women vs men, 90 s women vs men). Surprisingly, these 30 s exposed women ages 46–64 had up to 46% prevalence for psychiatric load, significantly higher than their 15 s counterparts (p < 0.01, 2021–2023 15 s vs 30 s).

After October 7th, (Fig. 1B) a significantly effect was observed in the 15 s group, in both men and women of all ages. In addition, a moderate yet significant increase was noted in other exposure areas. 15 s women aged 46–64 surpassed 30 s women in psychiatric load rates of 66% and 42% respectively (p < 0.001, 15 s vs 90 s; p < 0.001, 30 s vs 90 s, p < 0.01,15 s vs 30 s). A significant increase in women and men 19–45 and 46–64 in all shelter times, even in low exposure, is observed in a exposure gradient manner when comparing the baseline of % new cases of psychiatric load pre-Oct 7th vs post-Oct 7th (Fig. 1B, upper left, inset) within the same shelter time (15 s pre vs 15 s post, 19–45, 36% increase, p < 0.001; 19–45, 30 s pre vs 30 s post, 4% increase, p < 0.01; 90 s pre vs 90 s post, 2% increase, p < 0.001). Overall, 30 s women 46–64 were most vulnerable to mental health effects of CTS, while after added acute events, both men and women were vulnerable proportionately to the intensity level.

Next, we examined medication use in CTS to confirm the findings of the psychiatric load. Antidepressant prescriptions (Fig. 1C) were the most abundant of any drug class and were already consumed significantly higher in 15 s due to CTS, 5% in women 46–64 (2018, 15 s vs 90 s, p < 0.05). After October 7th (Fig. 1C left and center, insets), we observed a significant increase of 14% and 17% in the 19–45 and 46–64 age groups, respectively in 15 s women (15 s pre vs post, p < 0.0001) and an increase of 13% in 15 s men 46–64 (15 pre vs post, p < 0.001) (Fig. 1C, center, inset). 30 s and 90 s exposed groups had more modest, yet significant, increases within shelter time (30 s pre vs 30 s post, p < 0.05, 90 s pre vs. post, p < 0.01).

Use of anxiolytics (Fig. 1D, center) increased after Oct 7th in 15 s, 46–64 aged men (15 s pre vs. post, p < 0.05), and women (15 s pre vs. post, p < 0.001). 30 s women already had 3% higher rates from 2017–2022 (46–64, 65+ p < 0.05 30 s vs 90 s). For Anti-psychotics, hypnotics and see Supplementary Text and Figures.

**Fig. 2:**
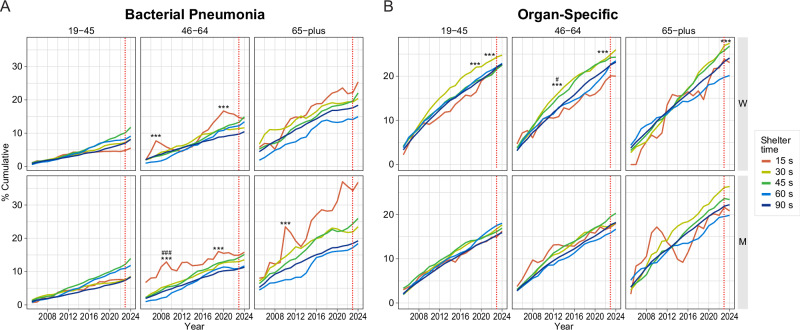
Prevalence rates of acute and chronic illnesses January 2005-August 2024. **A** Prevalence rates of bacterial pneumonia, in 15 s residents ages 46–64, were 6% higher in women (top, center panel) 2018–2022 p < 0.001,and men (lower, center panel) in 2019 (15 s vs 90 s, p < 0.001) with peak of 16% prevalence in 2019, and of up to 8% more than low exposure areas in 2008–2009 (15 s vs 90 s, p < 0.001 15 s vs 30 s, p < 0.001). **B**. Significantly higher prevalence rates of organ-specific autoimmune disorders, up to 26%, were observed in women 19–45 (top, left panel) and 46–64 (top, center panel) higher than both high and low exposed women in 2014–2016 (46–64, 30 s vs 90 s, p < 0.001, 15 s vs 30 s, p < 0.01, “#”) and in 2018–2019 (19–45, p < 0.001, 30 s vs 90 s).

Immune dysregulation was then assessed by examining prevalence rates of bacterial pneumonia and autoimmune disorders diagnosed per shelter time. Specifically, we looked at prevalence rates of bacterial pneumonia, a lung infection caused by an acute bacterial secondary infection in those with weakened immune systems. While older age groups are particularly vulnerable, 15 s 46–64 groups had higher prevalence rates that 90 s. 15 s women 46–64 (Fig. 2A top, center) had up to 5–6% higher prevalence, (2018–2022, 15 s vs 90 s, p < 0.0001), while 15 s men aged 46–64 (Fig. 2B, bottom, center), had a peak of 16% prevalence in 2019 (15 s vs 90 s, p < 0.0001), and of up to 8% higher than low exposure areas in 2008–2009 (15 s vs 90 s, p < 0.00000 15 s vs 30 s, p < 0.0001), demonstrating a vulnerability to this acute immune illness particularly in high exposed men.

Significantly higher prevalence of chronic, organ-specific autoimmune disorder (Fig. 2B) rates of up to 26% were observed 30 s women; in women 19–45 there were peaks in years 2018–2019 compared to 90 s (p < 0.001, 30 s vs 90 s) and in women 46–64 there were significant differences when compared to both 15 s and 90 s groups exposure in 2014–2016 (30 s vs 90 s, p < 0.001, 15 s vs 30 s, p < 0.01). Prevalence in women in general was significantly higher than men in 19–45 and 46–64 age groups in all years (2005–2024, p < 0.001, 15 s women vs men, 30 s women vs men, 90 s women vs men) indicating the sex-specific impact of moderate CTS on autoimmune disease in women. No acute effects of October 7th were observed in both acute and chronic illnesses, however, increases in prevalence may take years and not months to observe.

**Fig. 3:**
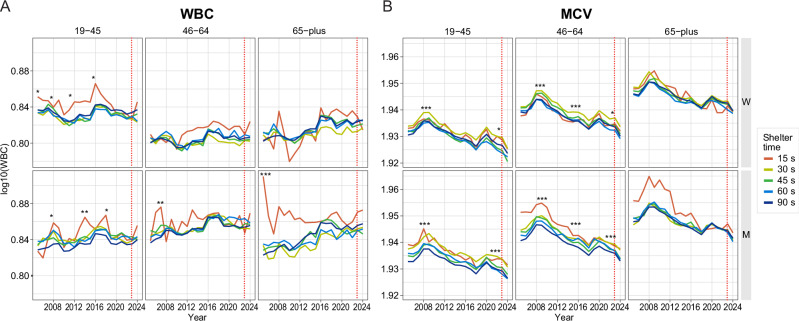
Standard laboratory tests. January 2005- August 2024 (**A**) WBC levels were most significantly elevated in years of escalation pre-October 7th in men 19–45 (15 s vs 90 s, 2008, p < 0.01, 2014-p < 0.001; 2018; p < 0.01, *d* = 0.29, bottom, left), men 46–64 (2007, p < 0.001, 15 s vs 90 s, *d* = 0.32, bottom, center panel) and men 65+ (2005 p < 0.001, *d* = 0.56, bottom, right panel). **B** MCV levels were significantly elevated in 30 s women pre-October 7th (30 s vs 90 s,19–45, 2005–2024, p < 0.01, top, right panel; 46–64, 2005–2024 p < 0.01, top center panel) with peak in 2010. Men, 30 s vs 90 s and women (19–45, 46–64, 30 s vs 90 s, 2005–2024, p < 0.001) with peaks 2009–2010, 2015, and 2022.

Standard laboratory blood test results were next examined to determine use as possible biomarkers of stress exposure and disease progression. All values were in the clinically healthy range, but white blood cells (WBC) levels (Fig. 3A) different significantly elevated in 15 s compared with other regions in earlier years of escalation. Specifically, WBC levels in men ages 46–64 in 15 s showed significant elevations and small effect size compared with 90 s group (15 s vs 90 s, 2007, p < 0.001, *d* = 0.32), but also men 65+ also had with significantly higher levels with a moderate effect size (15 s vs 90 s, 2005 p < 0.0000, *d* = 0.56) and men 19–45 had elevation in years with escalation in rocket fire (15 s vs 90 s, 2008, p < 0.01, 2014-p < 0.001; 2018; p < 0.01, *d* = 0.32). In contrast, mean corpuscular volume (MCV) levels were elevated significantly, in 30 s exposed women and 30 s men in all years in 19–45 and 46–64 compared to 90 s counterparts, (women: 30 s vs 90 s, 19–45, 2005–2024, p < 0.01; 46–64, 2005–2024 p < 0.01, *d* = 0.20 (men: 30 s; 19–45, 46–64, 30 s vs 90 s, 2005–2024, p < 0.001, *d* = 0.20) with peaks 2009–2010 and 2015. These results demonstrated exposure dependent alterations in these standard blood tests.

**Fig. 4:**
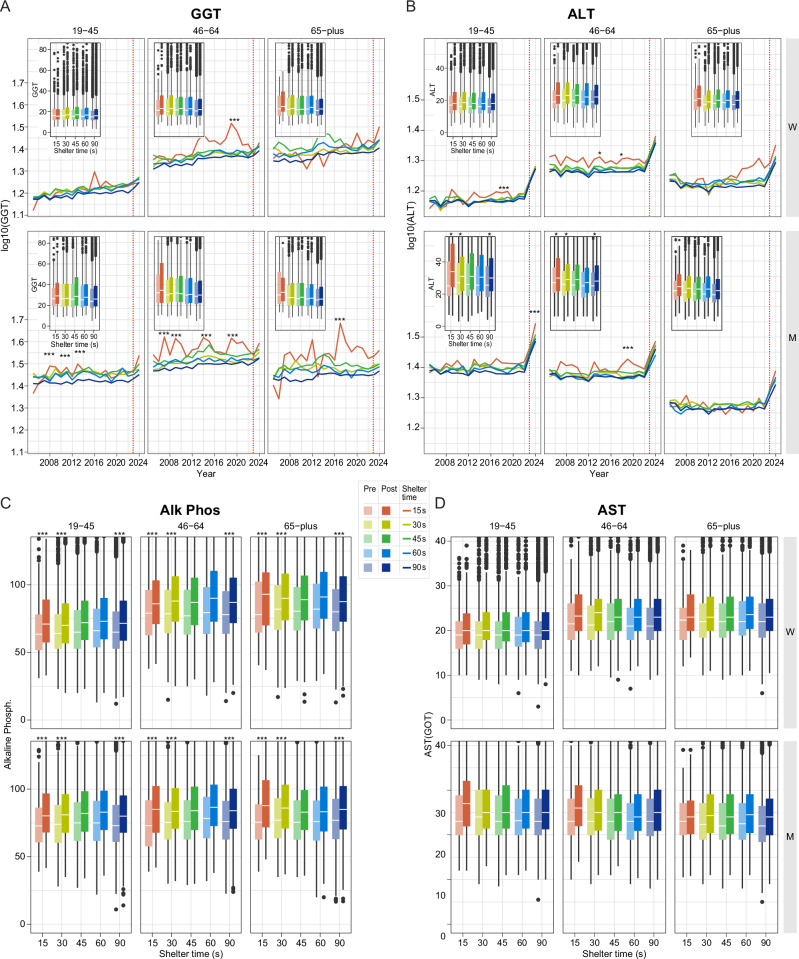
Liver Enzymes, Long-Term January 2005-August 2024 and Pre vs Post October 7th. **A** GGT were elevated chronically and signficantly specifically in high exposure men 46–64 (bottom, center) in years of escalation (2007, 15 s vs 90 s, p < 0.001; 2009, 15 s vs 90 s p < 0.001; 2019, 15 s vs 90 s, p < 0.001, *d* = 0.42), while women 46–64 showe*d* peak levels in 2019 (15 s vs 90 s, p < 0.001, *d* = 0.40). Men 19–45 elevations with a *d* = 0.27 (2008–2009, 15 s vs 90 s, p < 0.001; 2011, 15 s vs 90 s, p < 0.001; 2013–2014, 15 s vs 90 s, p < 0.01; 2022, 15 s vs 90 s, p < 0.001). Men 19–45 had highest post-October 7th levels (lower, left, inset, 15 s post vs 90 s post, 15 s post avg, 60.521, SD 13.5, 90 s post avg, 35.9, SD 0.47, p < 0.0001). **B** ALT were also chronically elevated in 15 s in both men (46–64, peak 2019 p < 0.001, *d* = 0.28, bottom, center panel) and in women 19–45 had particulary high elevation in 2016, (15 s vs 90 s p < 0.001, *d* = 0.37), while women 46–64 had elevations from from 2013–2022 (15 s vs 90 s p < 0.01). Post-October 7th, males 19–45 in all shelter times had elevations (p < 0.01, 15 s pre vs 15 s post, *d* = 0.20; 30 s pre vs 30 s post, 90 s pre vs 90 s with small *d* = < 0.20) (**C**) Alkaline Phosphate (Alk Phos) levels, also increased significantly pre vs post October 7th in every age group (insets), gender an*d* shelter time, even low exposure (men, 19–45, 46–64, 65+, p < 0.001,15 s pre vs 15 s post; p < 0.001 30 s pre vs 30 s post ; p < 0.001, *d up to* 0.35). **D** In AST had significant changes within each shelter time pre vs post October 7th in the same direction of elevation in all ages except 15 s males over age 65 + , with highest *d* = 0.2 in women ages 46–64 (p < 0.001, 30 pre vs 30 s post).

Overall liver enzymes were especially sensitive to exposure, each in a distinct manner. Gamma-glutamyl transferase (GGT) levels (Fig. 4A) were elevated chronically and significantly in both men and women with small to moderate effect size in high exposure areas despite being in a normal healthy range. In men in years of escalation there was a significant elevation compared to 90 s men in the 46–64 age group (2007, 15 s vs 90 s, p < 0.001; 2009, 15 s vs 90 s p < 0.001; 2019, 15 s vs 90 s, p < 0.001, up to *d* = 0.42), while 15 s women 46–64 had a 2019 peak compared with 90 s (15 s vs 90 s, p < 0.0001, *d* = 0.40). 15 s men 19–45 exhibited significant elevations compared with 90 s men (2008–2009, 15 s vs 90 s, p < 0.001; 2011, 15 s vs 90 s, p < 0.001; 2013–2014, 15 s vs 90 s, p < 0.01; 2022, 15 s vs 90 s, p < 0.001, up to *d* = 0.27). After acute exposure (Fig. 4A lower, left inset), 15 s men 19–45 had highest post-Oct 7^th^ levels (15 s post vs 90 s post, 15 s post avg, 60.521, SD 13.5, 90 s post avg, 35.9, SD 0.47, p < 0.0001).

Alanine transaminase (ALT) (Fig. 4B) was also chronically elevated prior to October 7th in 15 s women and 15 s men (men, 46–64, peak 2019 p < 0.001, *d* = 0.28). After the acute exposure on October 7th, there were significant changes in males 19–45 within each shelter times (15 s pre vs 15 s post, 30 s pre vs 30 s post, 90 s pre vs 90 s with *d* = 0.20). 15 s women 19–45 had a peak elevation in 2016 and small to moderate effect size compared with 90 s women, (15 s vs 90 s p < 0.001, *d* = 0.37), while 15 s women aged 46–64 had elevations from 2013–2022 (15 s vs 90 s p < 0.01). 15 s men aged 46–64, had peak significant elevation in 2019 (p < 0.001 15 vs 90 s, *d* = 0.28). After October 7th, both men and women also had significant elevations from baseline in all shelter times, specifically 30 s women aged 46–64 pre-Oct 7th compared with 30 s women post Oct 7th (p < 0.001, 30 s pre vs 30 s post, *d* = 0.25). Men of all shelter times aged 19–45 were significantly elevated from baseline levels in all shelter times (p < 0.01, 15 s pre vs 15 s post, *d* = 0.20, p < 0.01, 30 s pre vs 30 s post, 90 s pre vs 90 s, *d* = 0.2).

Alkaline Phosphate (Alk Phos) levels (Fig. 4C) also highly increase significantly pre- vs post-October 7th in every age group, sex and shelter time, even in 90 s exposure, with small to moderate effect size, *d* = 0.35, in men (19–45, 46–64, 65 + , p < 0.001,15 s pre vs 15 s post, (p < 0.001 30 s pre vs 30 s post; p < 0.001 90 s pre vs post).

Aspartate transferase (AST) had significant elevations within each shelter time pre- vs post-October 7th in both sexes (men: p < 0.01, 19–45, 46–64, 15 s pre vs 15 s post, 30 s pre vs post, 90 s pre vs post, *d* < 0.20; women: 46–64, p < 0.001, 30 s pre vs 30 s post, *d* = 0.20). Liver enzymes compared pre vs post Oct 7^th^ were significant in men in an exposure dependent gradient.

We next used generalized linear regression models to examine interactions between exposure, sex, and blood biomarkers in relation to diagnostic outcomes among adults aged 46–64 years, the group showing the greatest vulnerability to sex-specific disease patterns under differential exposure (Table 1).

**Table 1:** Generalized Linear Model of Factors associated with Psychiatric Load and Organ-Specific Autoimmune Disorders.

Factors	Psychiatric Load			Organ-Specific		
	estimate	OR	p.value	estimate	OR	p.value
(Intercept)	−0.237	0.789	0.000	−1.091	0.336	0.000
Shelter Time 15 s	1.899	**6.682**	0.000	−0.164	0.849	NS
Shelter Time 30 s	0.376	**1.457**	0.000	0.184	**1.202**	0.023
Sex: Male vs Female	−0.414	**0.661**	0.000	−0.177	**0.837**	0.001
WBC	0.097	**1.102**	0.000	−0.022	0.978	NS
GGT	−0.005	0.995	NS	0.028	1.028	NS
MCV	0.171	**1.186**	0.000	0.072	**1.075**	0.021
Alkaline Phosphate	0.007	1.007	NS	−0.056	0.946	NS
ALT	0.021	1.021	NS	0.015	1.015	NS
Shelter Time 15 s X Sex	−0.503	0.605	NS	−0.349	0.705	NS
Shelter Time 30 s X Sex	−0.254	**0.776**	0.046	−0.254	0.776	0.083 ~
Shelter Time 15 s X WBC	−0.468	**0.626**	0.048	0.320	1.377	NS
Shelter Time 30 s X WBC	0.044	1.045	NS	0.153	**1.166**	0.039
Shelter Time 15 s X GGT	0.237	1.268	NS	−0.459	**0.632**	0.087
Shelter Time 30 s X GGT	0.121	1.128	NS	−0.061	0.941	NS
Shelter Time 15 s X MCV	−0.206	0.814	NS	−0.089	0.915	NS
Shelter Time 30 s X MCV	−0.076	0.927	NS	−0.196	**0.822**	0.024
Shelter Time 15 s X Alk Phos	−0.520	0.594	NS	0.785	**2.193**	0.025
Shelter Time 30 s X Alk Phos	0.153	1.165	NS	−0.125	0.882	NS
Shelter Time 15 s X ALT	−0.028	0.972	NS	0.428	1.535	NS
Shelter Time 30 s X ALT	−0.100	0.905	NS	−0.062	0.940	NS
Shelter Time 90 s X Sex X WBC	−0.043	0.958	NS	−0.074	**0.929**	0.077~
Shelter Time 15 s X Sex X WBC	0.610	**1.840**	0.049	−0.368	0.692	NS
Shelter Time 30 s X Sex X WBC	−0.017	0.983	NS	−0.104	0.901	NS
Shelter Time 90 s X Sex X GGT	0.056	1.057	NS	−0.036	0.965	NS
Shelter Time 15 s X Sex X GGT	−0.274	0.760	NS	0.569	1.767	NS
Shelter Time 30 s X Sex X GGT	−0.140	0.869	NS	−0.042	0.959	NS
Shelter Time 90 s X Sex X MCV	−0.123	**0.885**	0.004	−0.142	**0.868**	0.004
Shelter Time 15 s X Sex X MCV	0.103	1.109	NS	−0.245	0.783	NS
Shelter Time 30 s X Sex X MCV	0.103	1.109	NS	0.013	1.013	NS
Shelter Time 90 s X Sex X Alk Phos	0.001	1.001	NS	0.049	1.050	NS
Shelter Time 15 s X Sex X Alk Phos	0.803	2.231	0.091 ~	−1.166	**0.312**	0.015
Shelter Time 30 s X Sex XAlk Phos	−0.090	0.914	NS	0.204	1.227	NS
Shelter Time 90 s X Sex X ALT	−0.084	**0.919**	0.047	−0.008	0.992	NS
Shelter Time 15 s X Sex X ALT	0.001	1.001	NS	−0.126	0.882	NS
Shelter Time 30 s X Sex X ALT	0.014	1.014	NS	0.132	1.141	NS

For psychiatric load, high exposure (15 s) was associated with substantially higher odds compared with low exposure (90 s), a more than a six-fold increase (OR = 6.68, p < 0.000), while moderate exposure (30 s) was associated with 46% higher odds (OR = 1.46, p < 0.000). Overall, men had lower odds of psychiatric diagnoses than women (OR = 0.66, p < 0.05), an effect that was particularly evident in the 30 s exposure zone (OR = 0.78, p < 0.05). Higher WBC counts were associated with increased odds of psychiatric diagnoses overall (OR = 1.10, p < 0.000), and interaction analyses indicated that men in the 15 s zone with elevated WBC had 84% higher odds of psychiatric load compared with the reference group (OR = 1.84, p < 0.05).

For organ-specific autoimmune disease, moderate exposure (30 s) was associated with 20% higher odds relative to low exposure (90 s) (OR = 1.20, p < 0.05). Higher MCV was associated with a modest but statistically significant increase in odds (OR = 1.07, p < 0.05), whereas lower MCV in the 30 s zone was associated with reduced odds (OR = 0.82, p < 0.05). Elevated WBC in the 30 s zone was also associated with higher odds of autoimmune disease (OR = 1.17, p < 0.05). Higher GGT levels showed a trend toward reducing odds in the 15 s zone (OR = 0.63, p = 0.09).

The strongest associations were observed for alkaline phosphatase (ALP) in which elevated ALP in the 15 s zone was associated with more than two-fold higher odds of autoimmune disease overall (OR = 2.19, p = 0.02). Sex-specific interaction terms indicated increased odds among women with elevated ALP in the 15 s zone, whereas men with elevated ALP in the same exposure zone exhibited substantially lower odds of autoimmune disease relative to women (OR = 0.31, p = 0.02).

**Fig. 5:**
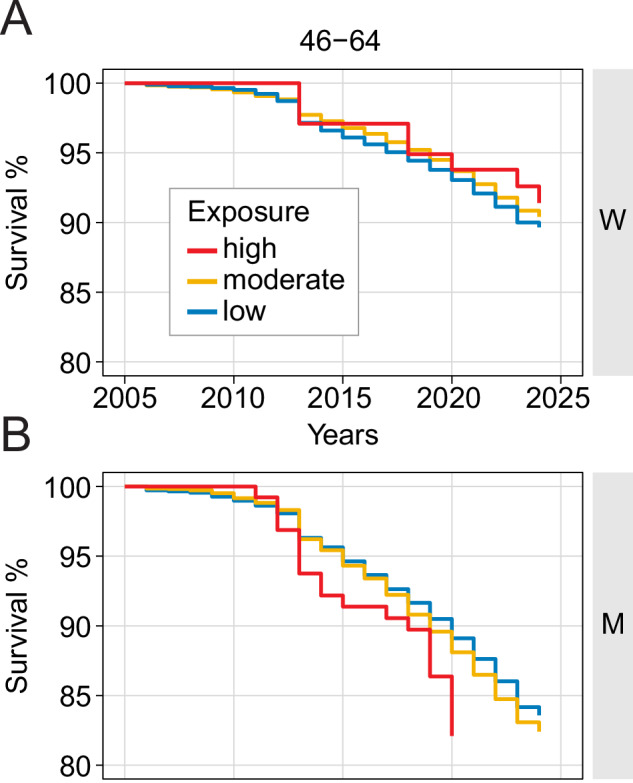
Survival Curve in the 46–64 age group. Survival Curve 2005–2023 in the vulnerable 46–64 age group (**A**) Females in moderately exposed (30 s, 45 s 60 s) regions had a slight increase of mortality compared to low exposed (90 s), 14% (p < 0.01) and no increased risk due to high exposure. **B** Men living in high exposed areas had a significantly 88% increased risk of mortality (HR = 1.88, p < 0.001; CI, 1.20–2.60, 15 s vs 90 s), while moderately exposed males (30 s, 45 s, 60 s) had only a 12% increased risk (HR = 1.12, p < 0.01; CI, 1.00–1.27). Comparing men to women, 15 s men had a 374% increased risk of mortality (HR = 3.74, p < 0.001; CI: 1.54–9.1), while moderate exposed men had a 178% increased risk in mortality (HR = 1.78 p < 0.000; CI :1.43–2.2) and low exposed men had a 185% increased risk in mortality (HR = 1.85, p < 0.000; CI:1.72–1.99).

Due to vulnerability to both high and moderate CTS from 2005–2023 and considering both psychiatric and physical manifestations of CTS, we examined survival rates in the affected age groups, 46–64, in both women and men. The results were highly significant showing that men ages 46–64 (Fig. 5B) living in 15 s areas, had a significantly 88% increased risk of death (HR = 1.88, p < 0.001; CI, 1.20–2.60), compared with moderately (30 s, 45 s, 60 s) exposed men had only a 12% increased risk (HR = 1.12, p < 0.001; CI, 1.05–1.24). Women in moderately exposed regions (30 s, 45 s, 60 s) had an increase of 14% (HR = 1.12, p < 0.01; CI, 1.00–1.27) and no increased risk due to 15 s exposure (Fig. 5A). Comparing men to women, 15 s men had a 374% increased risk of mortality (HR = 3.74, p < 0.001; CI: 1.54–9.1), while moderate exposed men had a 178% increased risk in mortality (HR = 1.78 p < 0.000; CI :1.43–2.2) and low exposed men had a 185% increased risk in mortality (HR = 1.85,p < 0.000; CI:1.72–1.99). These results highlight that high exposure in men was a robust sex-specific risk factor for mortality when compared to women or men of other shelter times.

## Discussion

Based on the large epidemiological civilian cohort, our findings demonstrate that CTS and additional acute trauma exert exposure-dependent effects on mental health, immune function, liver biomarkers, and mortality. The observed outcomes varied systematically by the exposure intensity, age and interactions of sex, underscoring the complex interplay between biological vulnerability to CTS.

Psychiatric morbidity increased across all exposure groups during the years of CTS, with the highest baseline prevalence observed in women from the 30 s region. Following the October 7th attacks, however, psychiatric morbidity rose in an intensity-dependent manner, suggesting that chronic stress may prime individuals for heightened vulnerability when faced with acute trauma [4, 33–35]. Notably, 15 s women, who had previously shown relative resilience in this cohort examination and others [36], exhibited marked increases in psychiatric diagnoses post-attack, consistent with the idea of stress sensitization after a traumatic exposure [37].

Medication data revealed complementary patterns. Prior to October 7th, 15 s women had relatively lower psychiatric diagnosis rates but higher antidepressant use, whereas 30 s residents showed greater anxiolytic use. These distinctions suggest differences in help-seeking behaviors and treatment pathways, reflecting both physiological stress coping responses and subjective differences in distress type i.e. sleeping disturbances.

Immune-related vulnerability was also observed, underscoring the role of stress in immune suppression and increased infection susceptibility and sex-specific manifestations. Elevated numbers of bacterial pneumonia cases spiked in years of escalation, and elevated WBC levels particularly in 15 s areas were observed in men. By contrast, organ-specific autoimmune disorders, representing long-term consequences of CTS, were observed in 30 s women, demonstrating immune changes over time.

Additionally, liver enzymes were observed as stress sensitive. GGT and Alkaline Phosphate levels were particularly elevated in acute stress post-October 7^th^ in 19–45 aged men. These elevations could be attributed to unhealthy coping to stress via consumption of alcohol, drugs, and processed food [38, 39], of which can initiate metabolic disorders [40, 41]. Liver responses to unhealthy lifestyle choices together coupled the long-terms effect of glucocorticoids elevations could synergistically impact the progression of fatty liver disease [42] and liver inflammation [43].

A central finding of this study is the sex-specific vulnerability of adults aged 46–64. Women in this age group were particularly prone to psychiatric morbidity and autoimmune conditions under moderate CTS exposure, whereas men showed stronger more robust changes in mortality. The vulnerability of the 46–64 group likely reflects the convergence of biological and psychosocial stressors. For women, menopause and estrogen decline, cause heightening of HPA-axis reactivity and pro-inflammatory responses, predisposing to psychiatric morbidity and autoimmunity [44, 45]. For men, similarly cortisol-mediated suppression of testosterone may impair cardiovascular and immune resilience [46, 47]. Sex differences in stress-related immune dysregulation predisposes to infections, a pattern supported by our finding of elevated WBC in younger men and bacterial pneumonia in older men [48, 49]. Additionally, men may underreport psychiatric distress [50], which may explain the lower observed psychiatric load but does not negate underlying physiological burden of stress, due to the convergence of elevated cortisol, reduced testosterone, and rising pro-inflammatory cytokines, all of which also contribute to depression, in addition to immune and cardiovascular disease [51–53]. Telomere shortening may represent the biological endpoint of perceived stress, chronic stress exposure, depression, [13, 14], accelerating cellular aging and promoting earlier disease progression. This process could help explain why excess mortality and morbidity appear most prominently in the 46–64 age group rather than in those 65 + , effectively shifting age-related vulnerability to an earlier-than-expected stage of life.

Overall, this large, epidemiological, longitudinal, civilian, digital medical record study of nearly two decades, reveal subgroup-specific patterns via a lifespan approach, stratifying by age and sex. By integrating diagnoses, biomarkers, and mortality outcomes, we provide a multidimensional picture of stress-related health trajectories. Some limitations included a small sample size in the highest-exposure (15 s) group, which constrained granularity of stratification. Additionally, although blood test values varied significantly across exposure levels and had small to moderate effect size, but most remained within clinical reference ranges, indicating subtle long-term shifts that may nonetheless precede disease onset.

While self-reported measures such as the Perceived Stress Scale [54] indeed capture every individual experiencing subjective distress, prevalence estimates used in this study derived from clinically verified diagnoses and prescription data offer a more conservative, yet robust assessment of stress-related morbidity over time. This methodology, objective and evidence-based, highlights population-level mental health outcomes by identifying individuals in sufficient distress to seek medical attention and thus bridging the gap between subjective distress and clinically recognized disease. Additional social factors might also impact exposure-dependent outcomes. While community or social cohesion measures [55] and socioeconomic factors influence psychological distress, disease rates and mortality [56], the dataset was limited in our ability to draw conclusions on these factors, though it has been shown that personal exposure has a high impact on psychiatric outcomes [57]. Finally, we set out to examine, acute and chronic stress exposures, which cannot be fully disentangled. All residents experienced CTS, but exposure intensity varied by shelter zone, shaping the differential impact of acute escalations.

Our results, however, still suggest that subtle biological changes i.e WBC, MCV, and liver enzymes may serve as early biomarkers of stress-related vulnerability. Preventive strategies including early laboratory screening and targeted interventions to reduce pro-inflammatory cytokines such as cognitive behavioral therapy [58] may help modify disease trajectories in high and moderate stress-exposed populations.

The authors declare that they have no known competing financial interests or personal relationships that could have appeared to influence the work reported in this paper.

## Supplementary information

Supplementary Tables

Supplementary Text

Supplementary Figure 1

## Data Availability

The data that support the findings of this study were obtained from Leumit Health Services under a data use agreement and are not publicly available due to privacy and legal restrictions. Data may be available upon reasonable request and with permission of the data custodians.
