# Supplementary material for: Intensity and exposure proximity as determinants of differential stress-related health outcomes
Source: Mol Psychiatry. 2026 Mar 6;31(7):3955–64. doi: 10.1038/s41380-026-03515-5 (PMC13269130; doi:10.1038/s41380-026-03515-5)

Supplementary Table 1: Demographic Information

Supplementary Table 2: Escalations and Relative Exposure between shelter times. The number of Sirens per shelter time during escalations, proportions of sirens per total siren number (%) and type of exposure during that time period.


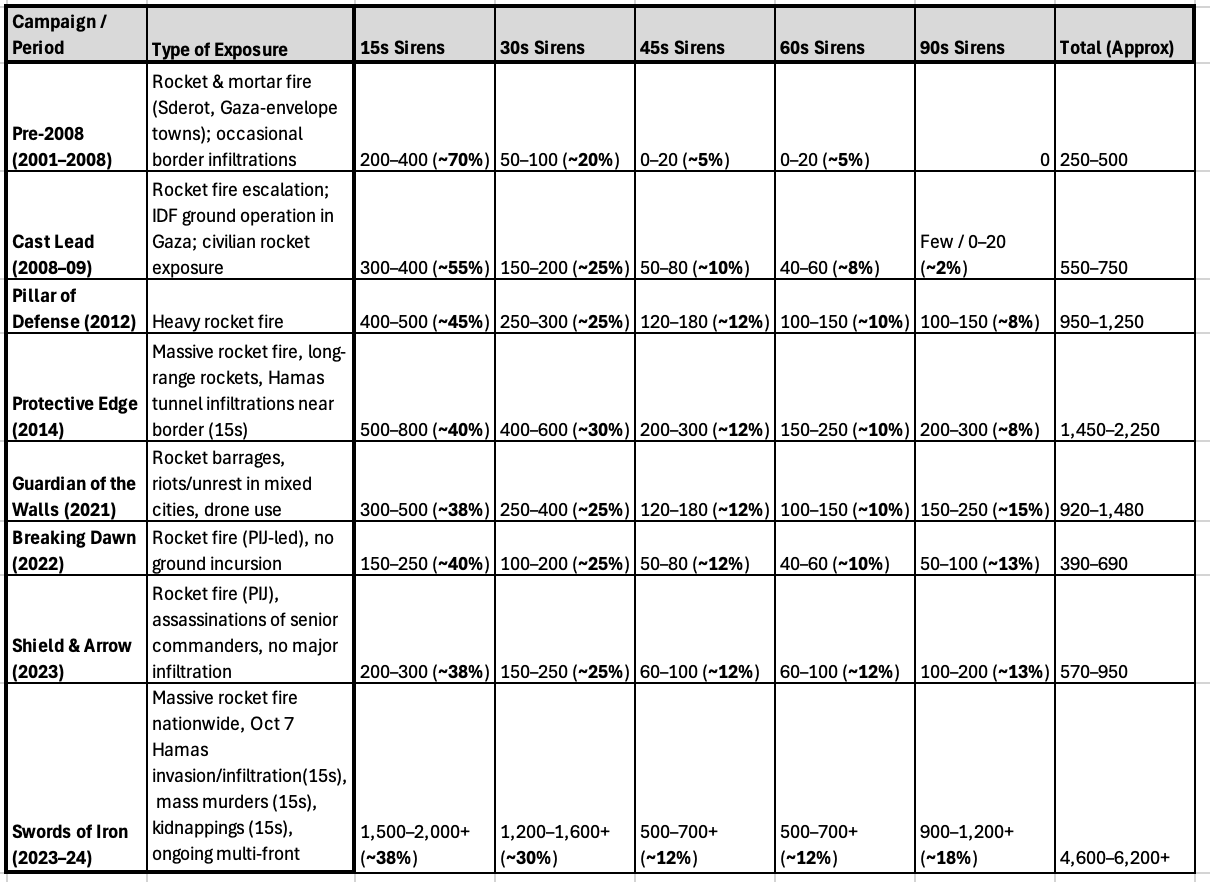


Supplementary Table 3: ICD-9 & ICD-10 codes used for Psychiatric Load

| ICD 9/10 code | Name of diagnosis | category within psychiatric load |
| --- | --- | --- |
| F43.2 | ADJUSTMENT DISORDERS | Adjustment |
| F43.20 | ADJUSTMENT DISORDERS, BRIEF DEPRESSIVE REACTION | Adjustment |
| F43.8 | ADJUSTMENT DISORDERS, OTHER REACTION TO SEVERE STRESS | Adjustment |
| F43.21 | ADJUSTMENT DISORDERS, PROLONGED DEPRESSIVE REACTION | Adjustment |
| F43.9 | ADJUSTMENT DISORDERS, REACTION TO SEVERE STRESS, UNSPECIFIED | Adjustment |
| F43.25 | ADJUSTMENT DISORDERS, WITH MIXED DISTURBANCE OF EMOTIONS AND CONDUCT | Adjustment |
| F43.28 | ADJUSTMENT DISORDERS, WITH OTHER SPECIFIED PREDOMINANT SYMPTOMS | Adjustment |
| F43.24 | ADJUSTMENT DISORDERS, WITH PREDOMINANT DISTURBANCE OF CONDUCT | Adjustment |
| F43.23 | ADJUSTMENT DISORDERS, WITH PREDOMINANT DISTURBANCE OF OTHER EMOTIONS | Adjustment |
| 309 | ADJUSTMENT REACTION | Adjustment |
| 309.24 | ADJUSTMENT REACTION WITH ANXIOUS MOOD | Adjustment |
| 309.0 | ADJUSTMENT REACTION WITH BRIEF DEPRESSIVE REACTION | Adjustment |
| 309.4 | ADJUSTMENT REACTION WITH MIXED DISTURBANCE OF EMOTIONS AND CONDUCT | Adjustment |
| 309.28 | ADJUSTMENT REACTION WITH MIXED EMOTIONAL FEATURES | Adjustment |
| 309.82 | ADJUSTMENT REACTION WITH PHYSICAL SYMPTOMS | Adjustment |
| 309.3 | ADJUSTMENT REACTION WITH PREDOMINANT DISTURBANCE OF CONDUCT | Adjustment |
| 309.2 | ADJUSTMENT REACTION WITH PREDOMINANT DISTURBANCE OF OTHER EMOTIONS | Adjustment |
| 309.1 | ADJUSTMENT REACTION WITH PROLONGED DEPRESSIVE REACTION | Adjustment |
| 309.83 | ADJUSTMENT REACTION WITH WITHDRAWAL | Adjustment |
| 309.22 | EMANCIPATION DISORDER OF ADOLESCENCE AND EARLY ADULT LIFE | Adjustment |
| 309.29 | OTHER ADJUSTMENT REACTIONS WITH PREDOMINANT DISTURBANCE OF OTHER EMOTIONS | Adjustment |
| 309.8 | OTHER SPECIFIED ADJUSTMENT REACTIONS | Adjustment |
| 309.89 | OTHER SPECIFIED ADJUSTMENT REACTIONS | Adjustment |
| F43.1 | POST-TRAUMATIC STRESS DISORDER[PTSD] | Adjustment |
| 309.81 | PROLONGED POSTTRAUMATIC STRESS DISORDER-PTSD | Adjustment |
| 309.21 | SEPARATION ANXIETY DISORDER | Adjustment |
| 309.23 | SPECIFIC ACADEMIC OR WORK INHIBITION | Adjustment |
| 309.9 | UNSPECIFIED ADJUSTMENT REACTION | Adjustment |
| F40.01 | AGORAPHOBIA, WITH PANIC DISORDER | Anxiety |
| F40.00 | AGORAPHOBIA, WITHOUT PANIC DISORDER | Anxiety |
| F41.9 | ANXIETY DISORDER, UNSPECIFIED | Anxiety |
| 300.0 | ANXIETY STATES | Anxiety |
| F93 | EMOTIONAL DISORDERS WITH ONSET SPECIFIC TO CHILDHOOD | Anxiety |
| F41.1 | GENERALIZED ANXIETY DISORDER | Anxiety |
| F42 | OBSESSIVE-COMPULSIVE DISORDER | Anxiety |
| 300.3 | OBSESSIVE-COMPULSIVE DISORDERS [OCD] | Anxiety |
| F41 | OTHER ANXIETY DISORDERS | Anxiety |
| F40.8 | OTHER PHOBIC ANXIETY DISORDERS | Anxiety |
| F41.8 | OTHER SPECIFIED ANXIETY DISORDERS SUCH AS MIXED ANXIETY-DEPRESSIVE DISORDER | Anxiety |
| F41.0 | PANIC DISORDER [EPISODIC PAROXYSMAL ANXIETY] | Anxiety |
| F93.1 | PHOBIC ANXIETY DISORDER OF CHILDHOOD | Anxiety |
| F40.9 | PHOBIC ANXIETY DISORDER, UNSPECIFIED | Anxiety |
| 300.2 | PHOBIC DISORDERS | Anxiety |
| F93.0 | SEPARATION ANXIETY DISORDER OF CHILDHOOD | Anxiety |
| F93.3 | SIBLING RIVALRY DISORDER | Anxiety |
| F93.2 | SOCIAL ANXIETY DISORDER OF CHILDHOOD | Anxiety |
| F40.1 | SOCIAL PHOBIAS | Anxiety |
| F40.2 | SPECIFIC (ISOLATED) PHOBIAS | Anxiety |
| 311 | DEPRESSIVE DISORDER, NOT ELSEWHERE CLASSIFIED | Depression |
| F32.9 | DEPRESSIVE EPISODE, UNSPECIFIED | Depression |
| 296.36 | MAJOR DEPRESSIVE AFFECTIVE DISORDER, RECURRENT EPISODE, IN FULL REMISSION | Depression |
| 296.36 | MAJOR DEPRESSIVE AFFECTIVE DISORDER, RECURRENT EPISODE, IN FULL REMISSION | Depression |
| 296.35 | MAJOR DEPRESSIVE AFFECTIVE DISORDER, RECURRENT EPISODE, IN PARTIAL OR UNSPECIFIED REMISSION | Depression |
| 296.35 | MAJOR DEPRESSIVE AFFECTIVE DISORDER, RECURRENT EPISODE, IN PARTIAL OR UNSPECIFIED REMISSION | Depression |
| 296.31 | MAJOR DEPRESSIVE AFFECTIVE DISORDER, RECURRENT EPISODE, MILD DEGREE | Depression |
| 296.31 | MAJOR DEPRESSIVE AFFECTIVE DISORDER, RECURRENT EPISODE, MILD DEGREE | Depression |
| 296.32 | MAJOR DEPRESSIVE AFFECTIVE DISORDER, RECURRENT EPISODE, MODERATE DEGREE | Depression |
| 296.32 | MAJOR DEPRESSIVE AFFECTIVE DISORDER, RECURRENT EPISODE, MODERATE DEGREE | Depression |
| 296.34 | MAJOR DEPRESSIVE AFFECTIVE DISORDER, RECURRENT EPISODE, SEVERE DEGREE, SPECIFIED AS WITH PSYCHOTIC BEHAVIOR | Depression |
| 296.34 | MAJOR DEPRESSIVE AFFECTIVE DISORDER, RECURRENT EPISODE, SEVERE DEGREE, SPECIFIED AS WITH PSYCHOTIC BEHAVIOR | Depression |
| 296.33 | MAJOR DEPRESSIVE AFFECTIVE DISORDER, RECURRENT EPISODE, SEVERE DEGREE, WITHOUT MENTION OF PSYCHOTIC BEHAVIOR | Depression |
| 296.33 | MAJOR DEPRESSIVE AFFECTIVE DISORDER, RECURRENT EPISODE, SEVERE DEGREE, WITHOUT MENTION OF PSYCHOTIC BEHAVIOR | Depression |
| 296.30 | MAJOR DEPRESSIVE AFFECTIVE DISORDER, RECURRENT EPISODE, UNSPECIFIED DEGREE | Depression |
| 296.30 | MAJOR DEPRESSIVE AFFECTIVE DISORDER, RECURRENT EPISODE, UNSPECIFIED DEGREE | Depression |
| 296.26 | MAJOR DEPRESSIVE AFFECTIVE DISORDER, SINGLE EPISODE, IN FULL REMISSION | Depression |
| 296.25 | MAJOR DEPRESSIVE AFFECTIVE DISORDER, SINGLE EPISODE, IN PARTIAL OR UNSPECIFIED REMISSION | Depression |
| 296.21 | MAJOR DEPRESSIVE AFFECTIVE DISORDER, SINGLE EPISODE, MILD DEGREE | Depression |
| 296.22 | MAJOR DEPRESSIVE AFFECTIVE DISORDER, SINGLE EPISODE, MODERATE DEGREE | Depression |
| 296.24 | MAJOR DEPRESSIVE AFFECTIVE DISORDER, SINGLE EPISODE, SEVERE DEGREE, SPECIFIED AS WITH PSYCHOTIC BEHAVIOR | Depression |
| 296.23 | MAJOR DEPRESSIVE AFFECTIVE DISORDER, SINGLE EPISODE, SEVERE DEGREE, WITHOUT MENTION OF PSYCHOTIC BEHAVIOR | Depression |
| 296.20 | MAJOR DEPRESSIVE AFFECTIVE DISORDER, SINGLE EPISODE, UNSPECIFIED DEGREE | Depression |
| 296.3 | MAJOR DEPRESSIVE DISORDER, RECURRENT EPISODE | Depression |
| 296.3 | MAJOR DEPRESSIVE DISORDER, RECURRENT EPISODE | Depression |
| 296.2 | MAJOR DEPRESSIVE DISORDER, SINGLE EPISODE | Depression |
| F32.0 | MILD DEPRESSIVE EPISODE | Depression |
| F32.1 | MODERATE DEPRESSIVE EPISODE | Depression |
| 300.4 | NEUROTIC DEPRESSION | Depression |
| F32.8 | OTHER DEPRESSIVE EPISODES | Depression |
| F33.8 | OTHER RECURRENT DEPRESSIVE DISORDERS | Depression |
| F38.1 | OTHER RECURRENT MOOD (AFFECTIVE) DISORDERS | Depression |
| F34.0 | PERSISTENT MOOD (AFFECTIVE) DISORDERS, CYCLOTHYMIA | Depression |
| F34.1 | PERSISTENT MOOD (AFFECTIVE) DISORDERS, DYSTHYMIA | Depression |
| F33 | RECURRENT DEPRESSIVE DISORDER | Depression |
| F33.0 | RECURRENT DEPRESSIVE DISORDER, CURRENT EPISODE MILD | Depression |
| F33.1 | RECURRENT DEPRESSIVE DISORDER, CURRENT EPISODE MODERATE | Depression |
| F33.3 | RECURRENT DEPRESSIVE DISORDER, CURRENT EPISODE SEVERE WITH PSYCHOTIC SYMPTOMS | Depression |
| F33.2 | RECURRENT DEPRESSIVE DISORDER, CURRENT EPISODE SEVERE WITHOUT PSYCHOTIC SYMPTOMS | Depression |
| F33.4 | RECURRENT DEPRESSIVE DISORDER, CURRENTLY IN REMISSION | Depression |
| F33.9 | RECURRENT DEPRESSIVE DISORDER, UNSPECIFIED | Depression |
| F32.3 | SEVERE DEPRESSIVE EPISODE WITH PSYCHOTIC SYMPTOMS | Depression |
| F32.2 | SEVERE DEPRESSIVE EPISODE WITHOUT PSYCHOTIC SYMPTOMS | Depression |
| 303 | ACUTE ALCOHOLIC INTOXICATION | substance dependence |
| 303.01 | ACUTE ALCOHOLIC INTOXICATION IN ALCOHOLISM, CONTINUOUS DRINKING BEHAVIOR | substance dependence |
| 303.02 | ACUTE ALCOHOLIC INTOXICATION IN ALCOHOLISM, EPISODIC DRINKING BEHAVIOR | substance dependence |
| 303.03 | ACUTE ALCOHOLIC INTOXICATION IN ALCOHOLISM, IN REMISSION | substance dependence |
| 303 | ACUTE ALCOHOLIC INTOXICATION IN ALCOHOLISM, UNSPECIFIED DRINKING BEHAVIOR | substance dependence |
| 291.1 | ALCOHOL AMNESTIC SYNDROME | substance dependence |
| 291.82 | ALCOHOL INDUCED SLEEP DISORDERS | substance dependence |
| 291.81 | ALCOHOL WITHDRAWAL | substance dependence |
| 291.0 | ALCOHOL WITHDRAWAL DELIRIUM | substance dependence |
| 291.3 | ALCOHOL WITHDRAWAL HALLUCINOSIS | substance dependence |
| 291.00 | ALCOHOLIC DELIRIUM | substance dependence |
| 291.01 | DELIRIUM TREMENS | substance dependence |
| 304 | DRUG DEPENDENCE | substance dependence |
| 292 | DRUG PSYCHOSES | substance dependence |
| 292.0 | DRUG WITHDRAWAL SYNDROME | substance dependence |
| 292.83 | DRUG-INDUCED AMNESTIC SYNDROME | substance dependence |
| 292.81 | DRUG-INDUCED DELIRIUM | substance dependence |
| 292.82 | DRUG-INDUCED DEMENTIA | substance dependence |
| 292.12 | DRUG-INDUCED HALLUCINOSIS | substance dependence |
| 292.84 | DRUG-INDUCED ORGANIC AFFECTIVE SYNDROME | substance dependence |
| 292.11 | DRUG-INDUCED ORGANIC DELUSIONAL SYNDROME | substance dependence |
| 291.4 | IDIOSYNCRATIC ALCOHOL INTOXICATION | substance dependence |
| F19 | MENTAL AND BEHAVIOURAL DISORDERS DUE TO MULTIPLE DRUG USE AND USE OF OTHER PSYCHOACTIVE SUBSTANCES | substance dependence |
| F19.07 | MENTAL AND BEHAVIOURAL DISORDERS DUE TO MULTIPLE DRUG USE AND USE OF OTHER PSYCHOACTIVE SUBSTANCES, ACUTE INTOXICATION, PATHOLOGICAL INTOXICATION | substance dependence |
| F19.03 | MENTAL AND BEHAVIOURAL DISORDERS DUE TO MULTIPLE DRUG USE AND USE OF OTHER PSYCHOACTIVE SUBSTANCES, ACUTE INTOXICATION, WITH DELIRIUM | substance dependence |
| F19.6 | MENTAL AND BEHAVIOURAL DISORDERS DUE TO MULTIPLE DRUG USE AND USE OF OTHER PSYCHOACTIVE SUBSTANCES, AMNESIC SYNDROME | substance dependence |
| F19.2 | MENTAL AND BEHAVIOURAL DISORDERS DUE TO MULTIPLE DRUG USE AND USE OF OTHER PSYCHOACTIVE SUBSTANCES, DEPENDENCE SYNDROME | substance dependence |
| F19.25 | MENTAL AND BEHAVIOURAL DISORDERS DUE TO MULTIPLE DRUG USE AND USE OF OTHER PSYCHOACTIVE SUBSTANCES, DEPENDENCE SYNDROME, CONTINUOUS USE | substance dependence |
| F19.23 | MENTAL AND BEHAVIOURAL DISORDERS DUE TO MULTIPLE DRUG USE AND USE OF OTHER PSYCHOACTIVE SUBSTANCES, DEPENDENCE SYNDROME, CURRENTLY ABSTINENT, BUT RECEIVING TREATMENT AND AVERSIVE OR BLOCKING DRUGS | substance dependence |
| F19.26 | MENTAL AND BEHAVIOURAL DISORDERS DUE TO MULTIPLE DRUG USE AND USE OF OTHER PSYCHOACTIVE SUBSTANCES, DEPENDENCE SYNDROME, EPISODIC USE (DIPSOMANIA) | substance dependence |
| F19.1 | MENTAL AND BEHAVIOURAL DISORDERS DUE TO MULTIPLE DRUG USE AND USE OF OTHER PSYCHOACTIVE SUBSTANCES, HARMFUL USE | substance dependence |
| F19.8 | MENTAL AND BEHAVIOURAL DISORDERS DUE TO MULTIPLE DRUG USE AND USE OF OTHER PSYCHOACTIVE SUBSTANCES, OTHER MENTAL AND BEHAVIOURAL DISORDERS | substance dependence |
| F19.5 | MENTAL AND BEHAVIOURAL DISORDERS DUE TO MULTIPLE DRUG USE AND USE OF OTHER PSYCHOACTIVE SUBSTANCES, PSYCHOTIC DISORDER | substance dependence |
| F19.56 | MENTAL AND BEHAVIOURAL DISORDERS DUE TO MULTIPLE DRUG USE AND USE OF OTHER PSYCHOACTIVE SUBSTANCES, PSYCHOTIC DISORDER, MIXED | substance dependence |
| F19.51 | MENTAL AND BEHAVIOURAL DISORDERS DUE TO MULTIPLE DRUG USE AND USE OF OTHER PSYCHOACTIVE SUBSTANCES, PSYCHOTIC DISORDER, PREDOMINANTLY DELUSIONAL | substance dependence |
| F19.52 | MENTAL AND BEHAVIOURAL DISORDERS DUE TO MULTIPLE DRUG USE AND USE OF OTHER PSYCHOACTIVE SUBSTANCES, PSYCHOTIC DISORDER, PREDOMINANTLY HALLUCINATORY | substance dependence |
| F19.73 | MENTAL AND BEHAVIOURAL DISORDERS DUE TO MULTIPLE DRUG USE AND USE OF OTHER PSYCHOACTIVE SUBSTANCES, RESIDUAL AND LATE-ONSET PSYCHOTIC DISORDER, DEMENTIA | substance dependence |
| F19.9 | MENTAL AND BEHAVIOURAL DISORDERS DUE TO MULTIPLE DRUG USE AND USE OF OTHER PSYCHOACTIVE SUBSTANCES, UNSPECIFIED MENTAL AND BEHAVIOURAL DISORDER | substance dependence |
| F19.3 | MENTAL AND BEHAVIOURAL DISORDERS DUE TO MULTIPLE DRUG USE AND USE OF OTHER PSYCHOACTIVE SUBSTANCES, WITHDRAWAL STATE | substance dependence |
| 291.90 | MENTAL AND BEHAVIOURAL DISORDERS DUE TO USE OF ALCOHOL | substance dependence |
| F10 | MENTAL AND BEHAVIOURAL DISORDERS DUE TO USE OF ALCOHOL | substance dependence |
| F10.0 | MENTAL AND BEHAVIOURAL DISORDERS DUE TO USE OF ALCOHOL, ACUTE INTOXICATION | substance dependence |
| F10.07 | MENTAL AND BEHAVIOURAL DISORDERS DUE TO USE OF ALCOHOL, ACUTE INTOXICATION, PATHOLOGICAL INTOXICATION | substance dependence |
| F10.6 | MENTAL AND BEHAVIOURAL DISORDERS DUE TO USE OF ALCOHOL, AMNESIC SYNDROME | substance dependence |
| F10.2 | MENTAL AND BEHAVIOURAL DISORDERS DUE TO USE OF ALCOHOL, DEPENDENCE SYNDROME | substance dependence |
| F10.25 | MENTAL AND BEHAVIOURAL DISORDERS DUE TO USE OF ALCOHOL, DEPENDENCE SYNDROME, CONTINUOUS USE | substance dependence |
| F10.23 | MENTAL AND BEHAVIOURAL DISORDERS DUE TO USE OF ALCOHOL, DEPENDENCE SYNDROME, CURRENTLY ABSTINENT, BUT RECEIVING TREATMENT AND AVERSIVE OR BLOCKING DRUGS | substance dependence |
| F10.26 | MENTAL AND BEHAVIOURAL DISORDERS DUE TO USE OF ALCOHOL, DEPENDENCE SYNDROME, EPISODIC USE (DIPSOMANIA) | substance dependence |
| F10.1 | MENTAL AND BEHAVIOURAL DISORDERS DUE TO USE OF ALCOHOL, HARMFUL USE | substance dependence |
| F10.5 | MENTAL AND BEHAVIOURAL DISORDERS DUE TO USE OF ALCOHOL, PSYCHOTIC DISORDER | substance dependence |
| F10.56 | MENTAL AND BEHAVIOURAL DISORDERS DUE TO USE OF ALCOHOL, PSYCHOTIC DISORDER, MIXED | substance dependence |
| F10.51 | MENTAL AND BEHAVIOURAL DISORDERS DUE TO USE OF ALCOHOL, PSYCHOTIC DISORDER, PREDOMINANTLY DELUSIONAL | substance dependence |
| F10.73 | MENTAL AND BEHAVIOURAL DISORDERS DUE TO USE OF ALCOHOL, RESIDUAL AND LATE-ONSET PSYCHOTIC DISORDER, DEMENTIA | substance dependence |
| F10.4 | MENTAL AND BEHAVIOURAL DISORDERS DUE TO USE OF ALCOHOL, WITHDRAWAL STATE WITH DELIRIUM | substance dependence |
| F12 | MENTAL AND BEHAVIOURAL DISORDERS DUE TO USE OF CANNABINOIDS | substance dependence |
| F12.2 | MENTAL AND BEHAVIOURAL DISORDERS DUE TO USE OF CANNABINOIDS, DEPENDENCE SYNDROME | substance dependence |
| F12.1 | MENTAL AND BEHAVIOURAL DISORDERS DUE TO USE OF CANNABINOIDS, HARMFUL USE | substance dependence |
| F14 | MENTAL AND BEHAVIOURAL DISORDERS DUE TO USE OF COCAINE | substance dependence |
| F14.2 | MENTAL AND BEHAVIOURAL DISORDERS DUE TO USE OF COCAINE, DEPENDENCE SYNDROME | substance dependence |
| F14.1 | MENTAL AND BEHAVIOURAL DISORDERS DUE TO USE OF COCAINE, HARMFUL USE | substance dependence |
| F16 | MENTAL AND BEHAVIOURAL DISORDERS DUE TO USE OF HALLUCINOGENS | substance dependence |
| F16.2 | MENTAL AND BEHAVIOURAL DISORDERS DUE TO USE OF HALLUCINOGENS, DEPENDENCE SYNDROME | substance dependence |
| F11 | MENTAL AND BEHAVIOURAL DISORDERS DUE TO USE OF OPIOIDS | substance dependence |
| F11.2 | MENTAL AND BEHAVIOURAL DISORDERS DUE TO USE OF OPIOIDS, DEPENDENCE SYNDROME | substance dependence |
| F11.1 | MENTAL AND BEHAVIOURAL DISORDERS DUE TO USE OF OPIOIDS, HARMFUL USE | substance dependence |
| F15.2 | MENTAL AND BEHAVIOURAL DISORDERS DUE TO USE OF OTHER STIMULANTS, INCLUDING CAFFEINE, DEPENDENCE SYNDROME | substance dependence |
| F15.1 | MENTAL AND BEHAVIOURAL DISORDERS DUE TO USE OF OTHER STIMULANTS, INCLUDING CAFFEINE, HARMFUL USE | substance dependence |
| F13 | MENTAL AND BEHAVIOURAL DISORDERS DUE TO USE OF SEDATIVES OR HYPNOTICS | substance dependence |
| F13.2 | MENTAL AND BEHAVIOURAL DISORDERS DUE TO USE OF SEDATIVES OR HYPNOTICS, DEPENDENCE SYNDROME | substance dependence |
| F13.1 | MENTAL AND BEHAVIOURAL DISORDERS DUE TO USE OF SEDATIVES OR HYPNOTICS, HARMFUL USE | substance dependence |
| F17 | MENTAL AND BEHAVIOURAL DISORDERS DUE TO USE OF TOBACCO | substance dependence |
| F17.1 | MENTAL AND BEHAVIOURAL DISORDERS DUE TO USE OF TOBACCO, HARMFUL USE | substance dependence |
| F15 | MENTAL AND BEHAVIOURAL DISORDERS DUE USE OF OTHER STIMULANTS, INCLUDING CAFFEINE | substance dependence |
| 304.0 | OPIOID TYPE DEPENDENCE | substance dependence |
| 291.2 | OTHER ALCOHOLIC DEMENTIA | substance dependence |
| 291.89 | OTHER ALCOHOLIC PSYCHOSIS | substance dependence |
| 303.9 | OTHER AND UNSPECIFIED ALCOHOL DEPENDENCE | substance dependence |
| 303.91 | OTHER AND UNSPECIFIED ALCOHOL DEPENDENCE, CONTINUOUS DRINKING BEHAVIOR | substance dependence |
| 303.92 | OTHER AND UNSPECIFIED ALCOHOL DEPENDENCE, EPISODIC DRINKING BEHAVIOR | substance dependence |
| 303.93 | OTHER AND UNSPECIFIED ALCOHOL DEPENDENCE, IN REMISSION | substance dependence |
| 303.9 | OTHER AND UNSPECIFIED ALCOHOL DEPENDENCE, UNSPECIFIED DRINKING BEHAVIOR | substance dependence |
| 291.8 | OTHER SPECIFIED ALCOHOLIC PSYCHOSIS | substance dependence |
| 292.8 | OTHER SPECIFIED DRUG-INDUCED MENTAL DISORDERS | substance dependence |
| 292.89 | OTHER SPECIFIED DRUG-INDUCED MENTAL DISORDERS | substance dependence |
| 292.1 | PARANOID AND/OR HALLUCINATORY STATES INDUCED BY DRUGS | substance dependence |
| 292.2 | PATHOLOGICAL DRUG INTOXICATION | substance dependence |
| 291.9 | UNSPECIFIED ALCOHOLIC PSYCHOSIS | substance dependence |
| 292.9 | UNSPECIFIED DRUG-INDUCED MENTAL DISORDER | substance dependence |
| F43.1 | POST-TRAUMATIC STRESS DISORDER[PTSD] | PTSD |
| 309.81 | PROLONGED POSTTRAUMATIC STRESS DISORDER-PTSD | PTSD |

Supplementary Table 4: List of Medications included in every medication class

| Drug Name | Drug Type | Drug Class |
| --- | --- | --- |
| ANAFRANIL 25 MG 30 TAB | Clomipramine | Antidepressants |
| SPRAVATO NS SP 28MG*2 | Esketamine | Antidepressants |
| SPRAVATO NS SP 28MG*3 | Esketamine | Antidepressants |
| DEPREXAN 25 MG 30 TAB | Desipramine | Antidepressants |
| ELATROL 25 MG 50 DRA | Amitriptyline | Antidepressants |
| ELATROLET 10 MG 50 DRAG | Amitriptyline | Antidepressants |
| GILEX 10 MG 30 CAP | Doxepin | Antidepressants |
| GILEX 25 MG 30 CAP | Doxepin | Antidepressants |
| MELODIL 10 MG 50 TABM | Maprotiline | Antidepressants |
| MELODIL 75 MG 20 TAB | Maprotiline | Antidepressants |
| LUDIOMIL 25 MG 10 AMP | Maprotiline | Antidepressants |
| MARONIL 10 MG 30 TAB | Clomipramine | Antidepressants |
| NARDIL 15 MG 100 TAB | Phenelzine | Antidepressants |
| NIAMID 100 MG 30 TAB | Nialamide | Antidepressants |
| NOVERIL 240 MG 20 TAB | Dibenzepin | Antidepressants |
| OPRIMOL 50 MG 30 DRAG | Opipramol | Antidepressants |
| PRIMONIL 25 MG 50 DRAG | Imipramine | Antidepressants |
| SURMONTIL 25 MG 50 TAB | Trimipramine | Antidepressants |
| SURMONTIL 100 MG 20 T | Trimipramine | Antidepressants |
| TRAZODIL 50 MG 30 TAB | Trazodone | Antidepressants |
| TRYPTAL 50 DRAG | Amitriptyline | Antidepressants |
| VICTORIL 80 MG 20 TAB | Dibenzepin | Antidepressants |
| MARONIL 25 MG 30 TAB | Clomipramine | Antidepressants |
| MELODIL 25 MG 30 TAB | Maprotiline | Antidepressants |
| MELODIL 50 MG 30 TAB | Maprotiline | Antidepressants |
| CIPRAMIL 40 MG 28 TAB | Citalopram | Antidepressants |
| TOFRANIL 25 MG 50 TAB | Imipramine | Antidepressants |
| TRAZODIL 100 MG 30 TAB | Trazodone | Antidepressants |
| FAVOXIL 50 MG 20 TAB | Fluvoxamine | Antidepressants |
| FAVOXIL 100 MG 10 TAB | Fluvoxamine | Antidepressants |
| BONSERIN 30 MG 20 TAB | Mianserin | Antidepressants |
| ANAFRANIL SR 75 MG 20 TAB | Clomipramine | Antidepressants |
| SEROXAT 20 MG 30 TAB | Paroxetine | Antidepressants |
| FAVOXIL 100 MG 30 TAB | Fluvoxamine | Antidepressants |
| FAVOXIL 50 MG 60 TAB | Fluvoxamine | Antidepressants |
| LUSTRAL 50 MG 28 TAB | Sertraline | Antidepressants |
| PRIZMA 20 MG 30 TAB | Fluoxetine | Antidepressants |
| EFEXOR 37.5 MG 30 TAB | Venlafaxine | Antidepressants |
| EFEXOR 75 MG 30 TAB | Venlafaxine | Antidepressants |
| FLUTINE 20 MG 30 CAP | Fluoxetine | Antidepressants |
| NORTYLIN 25 MG 30 TAB | Nortriptyline | Antidepressants |
| MARONIL SR 75 MG 20 TAB | Clomipramine | Antidepressants |
| CIPRAMIL 20 MG 28 TAB | Citalopram | Antidepressants |
| REMERON 45 MG 30 TAB | Mirtazapine | Antidepressants |
| FLUOXETINE4MG/1ML100 ML | Fluoxetine | Antidepressants |
| BE POSITIVE 30 CAPL | Tryptophan | Antidepressants |
| CYMBALTA 60 MG 28 TAB | Duloxetine | Antidepressants |
| CYMBALTA 30 MG 28 TAB | Duloxetine | Antidepressants |
| CITALOPRAM TEVA 20 MG 28T | Citalopram | Antidepressants |
| SEROXAT 20 MG 30 TAB | Paroxetine | Antidepressants |
| VENLAFAXINE XR 75 MG 30 C | Venlafaxine | Antidepressants |
| VENLAFAXINE XR 150 MG 30C | Venlafaxine | Antidepressants |
| VIEPAX XR 225 MG 30TAB | Venlafaxine | Antidepressants |
| FLUTINE 10 MG 30 C F | Fluoxetine | Antidepressants |
| EDRONAX 4 MG 60 TAB | Reboxetine Methan Sulfonate | Antidepressants |
| IXEL 25 MG 56 CAPS | Milnacipran | Antidepressants |
| IXEL 50 MG 56 CAPS | Milnacipran | Antidepressants |
| PAXXET 20 MG 30 TAB | Paroxetine | Antidepressants |
| PAXXET 30 MG 30 TAB | Paroxetine | Antidepressants |
| CIPRALEX 15 MG 28 TAB | Escitalopram | Antidepressants |
| VIEPAX XR 75 MG 30 TAB | Venlafaxine | Antidepressants |
| VIEPAX XR 150 MG 30 TAB | Venlafaxine | Antidepressants |
| LUSTRAL 100 MG 28 TAB | Sertraline | Antidepressants |
| RECITAL 20 MG 28 TAB | Citalopram | Antidepressants |
| REMOTIV 500 MG 30 TAB | Hyperici Herba | Antidepressants |
| EFEXOR 37.5 MG 29-G | Venlafaxine | Antidepressants |
| EFEXOR 75 MG 28 TAB | Venlafaxine | Antidepressants |
| EFEXOR 75 MG 28 29-G | Venlafaxine | Antidepressants |
| ESTO 15 MG 28 TAB | Escitalopram | Antidepressants |
| FLUOXETINE S 4MG/ML PREP | Fluoxetine | Antidepressants |
| DULOXETINE DR 30MG 30 CAP | Duloxetine | Antidepressants |
| DULOXETINE DR 60MG 30 CAP | Duloxetine | Antidepressants |
| RECITAL 40 MG 28 TAB | Citalopram | Antidepressants |
| CIPRALEX 10 MG 28 TAB | Escitalopram | Antidepressants |
| VIEPAX XR 150 MG NF 30TAB | Venlafaxine | Antidepressants |
| MIRO 30 MG 30 TAB | Mirtazapine | Antidepressants |
| MIRO 45 MG 30 TAB | Mirtazapine | Antidepressants |
| FLUOXETINE S 4MG/ML PREP | Fluoxetine | Antidepressants |
| PAROXETINE TEVA 20 MG 30T | Paroxetine | Antidepressants |
| VENLA 37.5 MG 28 TAB | Venlafaxine | Antidepressants |
| VENLA 75 MG 28 TAB | Venlafaxine | Antidepressants |
| PRIZMA SOL 4MG/ML120ML | Fluoxetine | Antidepressants |
| VIEPAX 37.5 MG 28 TAB | Venlafaxine | Antidepressants |
| VIEPAX 75 MG 28 TAB | Venlafaxine | Antidepressants |
| VIEPAX XR 75 MG 28 CAPL | Venlafaxine | Antidepressants |
| VIEPAX XR 150 MG 28 CAPL | Venlafaxine | Antidepressants |
| MIRTAZAPINE 15MG 28 TAB | Mirtazapine | Antidepressants |
| MIRTAZAPINE 30 MG 30 TAB | Mirtazapine | Antidepressants |
| MIRTAZAPINE 45 MG 30 TAB | Mirtazapine | Antidepressants |
| SERTRALINE TEVA 50 MG 30 | Sertraline | Antidepressants |
| REMOTIV 250 MG 60 TAB | Hyperici Herba | Antidepressants |
| LUSTRAL 100 MG 28 TAB | Sertraline | Antidepressants |
| LUSTRAL 50 MG 28 TAB | Sertraline | Antidepressants |
| DULOXETINE SK 30MG 28 CAP | Duloxetine | Antidepressants |
| DULOXETINE SK 60MG 28 CAP | Duloxetine | Antidepressants |
| DULOX TEVA 30 MG 30 CAP | Duloxetine | Antidepressants |
| DULOX TEVA 60 MG 30 CAP | Duloxetine | Antidepressants |
| SERTRALINE TAB 100 MG 30 | Sertraline | Antidepressants |
| SERENADA 50 MG 28 TAB | Sertraline | Antidepressants |
| SERENADA 100 MG 28 TAB | Sertraline | Antidepressants |
| MOBEMID 150 MG 30 TAB | Moclobemide | Antidepressants |
| PRIZMA FORTE 60 MG 20 TAB | Fluoxetine | Antidepressants |
| WELLBUTRIN XR 150 MG 30 T | Bupropion | Antidepressants |
| WELLBUTRIN XR 300 MG 30 T | Bupropion | Antidepressants |
| ESCITALOPRAM TEVA 10MG 30 | Escitalopram | Antidepressants |
| ESTO 10 MG 28 TAB | Escitalopram | Antidepressants |
| ESTO 20 MG 28 TAB | Escitalopram | Antidepressants |
| ELATROLET 10 MG 30 TAB | Amitriptyline | Antidepressants |
| ESCITALOPRAM TEVA 20MG 30 | Escitalopram | Antidepressants |
| BRINTELLIX 5 MG 28 TAB | Vortioxetine | Antidepressants |
| BRINTELLIX 10 MG 28 TAB | Vortioxetine | Antidepressants |
| BRINTELLIX 15 MG 28 TAB | Vortioxetine | Antidepressants |
| BRINTELLIX 20 MG 28 TAB | Vortioxetine | Antidepressants |
| CIPRALEX 20 MG 28 TAB | Escitalopram | Antidepressants |
| FLUVOXAMINE 100 MG 30 TAB | Fluvoxamine | Antidepressants |
| FLUVOXAMINE 50 MG 60 TAB | Fluvoxamine | Antidepressants |
| REMERON 30 MG 30 TAB | Mirtazapine | Antidepressants |
| TRIPT-OH 25MG 20 SACH 29G | Tryptophan | Antidepressants |
| FLUOXETINE 20MG 30CAP 29G | Fluoxetine | Antidepressants |
| NARDIL 15 MG 100 TAB 29-G | Phenelzine | Antidepressants |
| NARDIL 15 MG 60 TAB 29-G | Phenelzine | Antidepressants |
| NARDIL 15 MG 100 TAB 29-G | Phenelzine | Antidepressants |
| MARGYL 15 MG 30 TAB 29-G | Phenelzine | Antidepressants |
| NARDIL 15 MG 60 TAB 29-G | Phenelzine | Antidepressants |
| NARDIL 15MG 60TAB 29G RAZ | Phenelzine | Antidepressants |
| AMITRIPTYLINE 10MG 28 29G | Amitriptyline | Antidepressants |
| AMITRIPTYLINE 25MG 28 29G | Amitriptyline | Antidepressants |
| IMIPRAMINE 25 MG 28T 29-G | Imipramine | Antidepressants |
| IMIPRAMINE 25 MG 28T 29-G | Imipramine | Antidepressants |
| EDRONAX 4 MG 60 TAB 29-G | Reboxetine Methan Sulfonate | Antidepressants |
| FLUVOXAMIN10MG/ML100ML | Fluvoxamine | Antidepressants |
| SERTRALIN10MG/MLSYR100ML | Sertraline | Antidepressants |
| IMIPRAMINE 25MG 100T 29-G | Imipramine | Antidepressants |
| REMERON 30 MG 30 TAB 29-G | Mirtazapine | Antidepressants |
| PHENELZINE SUL 15MG T 29G | Phenelzine | Antidepressants |
| MIRTAZAPINE3.75MG/ML100ML | Mirtazapine | Antidepressants |
| NORTRIPTYLIN 25MG 100T 29 | Nortriptyline | Antidepressants |
| DESIPRAMINE 25MG 100 29G | Desipramine | Antidepressants |
| DESIPRAMINE 25MG 100 29G | Desipramine | Antidepressants |
| MILNACIPRAN 50MG 56C 29-G | Milnacipran | Antidepressants |
| MILNACIPRAN 25MG 56C 29-G | Milnacipran | Antidepressants |
| IMIPRAMINE 25MG 100T 29-G | Imipramine | Antidepressants |
| DOPARID 100 MG 30 TAB | Tiapride | Antipsychotics |
| ENTUMINE 40 MG 30 TAB | Clotiapine | Antipsychotics |
| FLUDECATE 12.5MG/0.5M | Fluphenazine | Antipsychotics |
| FLUDECATE 25MG/ML AMP 1ML | Fluphenazine | Antipsychotics |
| MAJEPTIL 10 MG 20 T | Thioproperazine | Antipsychotics |
| MELLERIL 25 MG 30 TAB | Thioridazine | Antipsychotics |
| MELLERIL 100 MG 30 T | Thioridazine | Antipsychotics |
| MODAL 2 ML 5 AMP | Sulpiride | Antipsychotics |
| MODAL 50 MG 30 CAP | Sulpiride | Antipsychotics |
| MODITEN 1 MG 100 TAB | Fluphenazine | Antipsychotics |
| MODITEN 5 MG 100 TAB | Fluphenazine | Antipsychotics |
| NEULEPTIL 10 MG 50 TAB | Periciazine | Antipsychotics |
| NEULEPTIL 4% DROPS | Periciazine | Antipsychotics |
| NOZINAN 25 MG 20 TAB | Levomepromazine | Antipsychotics |
| NOZINAN 100 MG 20 T | Levomepromazine | Antipsychotics |
| XEPLION 50 MG 1 VIAL | Paliperidone | Antipsychotics |
| XEPLION 75 MG 1 VIAL | Paliperidone | Antipsychotics |
| XEPLION 100 MG 1 VIAL | Paliperidone | Antipsychotics |
| XEPLION 150 MG 1 VIAL | Paliperidone | Antipsychotics |
| PERPHENAN 4 MG 30 TAB | Perphenazine | Antipsychotics |
| PERPHENAN 8 MG 20 TAB | Perphenazine | Antipsychotics |
| RIDAZIN 100 MG 30 TAB | Thioridazine | Antipsychotics |
| TERFLUZINE 10 MG 20 T | Trifluoperazine | Antipsychotics |
| CLOPIXOL DEPO 200MG/ML*10 | Zuclopenthixol | Antipsychotics |
| CLOPIXOL 10 MG 100 TAB | Zuclopenthixol | Antipsychotics |
| CLOPIXOL 25 MG 100 TAB | Zuclopenthixol | Antipsychotics |
| OLANZAPINE TEVA 5 MG 28 T | Olanzapine | Antipsychotics |
| OLANZAPINE TEVA 7.5MG 56T | Olanzapine | Antipsychotics |
| OLANZAPINE TEVA 10 MG 28T | Olanzapine | Antipsychotics |
| REAGILA 1.5 MG 30 TAB | Cariprazine | Antipsychotics |
| REAGILA 3 MG 30 TAB | Cariprazine | Antipsychotics |
| REAGILA 4.5 MG 30 TAB | Cariprazine | Antipsychotics |
| REAGILA 6 MG 30 TAB | Cariprazine | Antipsychotics |
| RIDAZIN 10 MG 40 TAB | Thioridazine | Antipsychotics |
| RIDAZIN 25 MG 30 TAB | Thioridazine | Antipsychotics |
| TAROCTYL 25 MG 50 TAB | Chlorpromazine | Antipsychotics |
| TAROCTYL 100 MG 50 TAB | Chlorpromazine | Antipsychotics |
| PERICATE 1 ML AMP | Haloperidol | Antipsychotics |
| HALIDOL 5MG TAB 30 | Haloperidol | Antipsychotics |
| CLOPIXOL 2 MG 50 TAB | Zuclopenthixol | Antipsychotics |
| CLOPIXOL 10 MG 50 TAB | Zuclopenthixol | Antipsychotics |
| CLOPIXOL 25 MG 50 TAB | Zuclopenthixol | Antipsychotics |
| CLOPIXOL DROPS | Zuclopenthixol | Antipsychotics |
| CLOPIXOL ACUPH 50 MG 1 ML | Zuclopenthixol | Antipsychotics |
| CLOPIXOL ACUPH 100 MG | Zuclopenthixol | Antipsychotics |
| CLOPIXOL DEPOT 200MG/ML-1 | Zuclopenthixol | Antipsychotics |
| MODAL FORTE 200 MG 40 TAB | Sulpiride | Antipsychotics |
| SAPHRIS 5 MG 60 TAB | Asenapine | Antipsychotics |
| SAPHRIS 10 MG 60 TAB | Asenapine | Antipsychotics |
| CLOPIXOL DEPOT 500 MG AMP | Zuclopenthixol | Antipsychotics |
| FLUANXOL 3 MG 100 TAB | Flupentixol | Antipsychotics |
| PERIDOR 1 MG 1000 T | Haloperidol | Antipsychotics |
| HALDOL DECN 100MG_ML 5AMP | Haloperidol | Antipsychotics |
| FLUANXOL DEPO 40 MG/2 ML | Flupentixol | Antipsychotics |
| FLUANXOL 1 MG 100 TAB | Flupentixol | Antipsychotics |
| RISPERDAL 2 MG 20 TAB | Risperidone | Antipsychotics |
| RISPERDAL 3 MG 20 TAB | Risperidone | Antipsychotics |
| RISPERDAL 4 MG 20 TAB | Risperidone | Antipsychotics |
| RISPERDAL 1 MG 20 TAB | Risperidone | Antipsychotics |
| TRUQUIL 15 MG 50 TAB | Chlorprothixene | Antipsychotics |
| TRUQUIL 5 MG 100 TAB | Chlorprothixene | Antipsychotics |
| TRUQUIL 50 MG 50 TAB | Chlorprothixene | Antipsychotics |
| ZYPREXA 5 MG 28 TAB | Olanzapine | Antipsychotics |
| ZYPREXA 7.5 MG 56 TAB | Olanzapine | Antipsychotics |
| ZYPREXA 10 MG 28 TAB | Olanzapine | Antipsychotics |
| ORAP FORTE 4 MG 20 TAB | Pimozide | Antipsychotics |
| SEMAP 20 MG 12 TAB 29-G | Penfluridol | Antipsychotics |
| HALDOL 15 ML DROPS | Haloperidol | Antipsychotics |
| HALDOL DECN 100MG_ML5 29G | Haloperidol | Antipsychotics |
| RISPERDAL CONS DELTOID 25 | Risperidone | Antipsychotics |
| RISPERDAL CONS DELTO 37.5 | Risperidone | Antipsychotics |
| RISPERDAL CONS DELTOID 50 | Risperidone | Antipsychotics |
| ZYPREXA VELOTAB 10MG 28 T | Olanzapine | Antipsychotics |
| ZYPREXA VELOTAB 15MG 28 T | Olanzapine | Antipsychotics |
| ZYPREXA VELOTAB 5 MG 28 T | Olanzapine | Antipsychotics |
| OLANZAPINE ODT 5 MG NEW | Olanzapine | Antipsychotics |
| OLANZAPINE ODT 10 MG NEW | Olanzapine | Antipsychotics |
| ZAPPA 5 MG 30 TAB | Olanzapine | Antipsychotics |
| ZAPPA 10 MG 30 TAB | Olanzapine | Antipsychotics |
| ZAPPA 7.5 MG 60 TAB | Olanzapine | Antipsychotics |
| RISPERIDEX 1 MG 30 TAB | Risperidone | Antipsychotics |
| RISPERIDEX 2 MG 30 TAB | Risperidone | Antipsychotics |
| RISPERIDEX 3 MG 30 TAB | Risperidone | Antipsychotics |
| OLANZAPINE TEVA ODT 10 MG | Olanzapine | Antipsychotics |
| OLANZAPINE TEVA ODT 5 MG | Olanzapine | Antipsychotics |
| ZYPADHERA 300 MG VIAL | Olanzapine | Antipsychotics |
| RISPERIDEX 4 MG 30 TAB | Risperidone | Antipsychotics |
| PERIDOR 1 MG 60 TAB | Haloperidol | Antipsychotics |
| PERIDOR 5 MG 60 TAB | Haloperidol | Antipsychotics |
| ZYPADHERA 210 MG VIAL | Olanzapine | Antipsychotics |
| ZYPADHERA 405 MG VIAL | Olanzapine | Antipsychotics |
| SEROQUELXR 150 MG 30 TAB | Quetiapine | Antipsychotics |
| LEPONEX 100 MG 50 TAB | Clozapine | Antipsychotics |
| LEPONEX 25 MG 50 TAB | Clozapine | Antipsychotics |
| RISPERDAL ORAL SOL 30 ML | Risperidone | Antipsychotics |
| HALDOL 5 MG 25 TAB | Haloperidol | Antipsychotics |
| LOZAPINE 25 MG 50 TAB | Clozapine | Antipsychotics |
| LOZAPINE 100 MG 50 TAB | Clozapine | Antipsychotics |
| RONEXINE 100MG 30TAB | Levomepromazine | Antipsychotics |
| RONEXINE 25 MG 30TAB | Levomepromazine | Antipsychotics |
| SEROQUEL 25 MG 30 TAB | Quetiapine | Antipsychotics |
| SEROQUEL 100 MG 30 TAB | Quetiapine | Antipsychotics |
| SEROQUEL 200 MG 30 TAB | Quetiapine | Antipsychotics |
| HALDOL 5 MG/1ML 5 AMP | Haloperidol | Antipsychotics |
| PERIDOR 10 MG 60 TAB | Haloperidol | Antipsychotics |
| PERICATE 100 MG/ML 5 AMP | Haloperidol | Antipsychotics |
| HALOPER 0.5 MG 60 TAB | Haloperidol | Antipsychotics |
| GEODON 20 MG 60 CAP | Ziprasidone | Antipsychotics |
| GEODON 40 MG 60 CAP | Ziprasidone | Antipsychotics |
| GEODON 60 MG 60 CAP | Ziprasidone | Antipsychotics |
| GEODON 80 MG 60 CAPS | Ziprasidone | Antipsychotics |
| SOLIAN 100 MG 30 TAB | Amisulpride | Antipsychotics |
| SOLIAN 400 MG 30 TAB | Amisulpride | Antipsychotics |
| PRIDE 400 MG 30 TAB | Amisulpride | Antipsychotics |
| ARIPLY 5 MG 28 TAB | Aripiprazole | Antipsychotics |
| ARIPLY 10 MG 28 TAB | Aripiprazole | Antipsychotics |
| ARIPLY 15 MG 28 TAB | Aripiprazole | Antipsychotics |
| ARIPLY 30 MG 28 TAB | Aripiprazole | Antipsychotics |
| PRIDE 100 MG 30 TAB | Amisulpride | Antipsychotics |
| PENFLURIDOL 20MG 12C PREP | Penfluridol | Antipsychotics |
| FANAPT 1 MG 60 TAB | Iloperidone | Antipsychotics |
| FANAPT 2 MG 60 TAB | Iloperidone | Antipsychotics |
| FANAPT 4 MG 60 TAB | Iloperidone | Antipsychotics |
| FANAPT 6 MG 60 TAB | Iloperidone | Antipsychotics |
| FANAPT 8 MG 60 TAB | Iloperidone | Antipsychotics |
| ZAPPA ODT 5 MG 30 TAB | Olanzapine | Antipsychotics |
| ZAPPA ODT 10 MG 30 TAB | Olanzapine | Antipsychotics |
| QUETIAPINE TEVA 25MG 30T | Quetiapine | Antipsychotics |
| QUETIAPINE TEVA 100MG 30T | Quetiapine | Antipsychotics |
| QUETIAPINE TEVA 200MG 30T | Quetiapine | Antipsychotics |
| QUETIAPINE TEVA 300MG 30T | Quetiapine | Antipsychotics |
| HALOPER 5 MG 60 TAB | Haloperidol | Antipsychotics |
| HALOPER 10 MG 60 TAB | Haloperidol | Antipsychotics |
| FANAPT TITRATION PACK | Iloperidone | Antipsychotics |
| ABILIFY MAINTENA 300MG SR | Aripiprazole | Antipsychotics |
| RISPOND 1 MG 30 TAB | Risperidone | Antipsychotics |
| RISPOND 2 MG 30 TAB | Risperidone | Antipsychotics |
| RISPOND 3 MG 30 TAB | Risperidone | Antipsychotics |
| RISPOND 4 MG 30 TAB | Risperidone | Antipsychotics |
| RISPERDAL CONSTA 25 MG | Risperidone | Antipsychotics |
| RISPERDAL CONSTA 50 MG | Risperidone | Antipsychotics |
| RISPERDAL CONSTA 37.5 MG | Risperidone | Antipsychotics |
| HALDOL 2MG/ML DROPS 30 ML | Haloperidol | Antipsychotics |
| REXULTI 1 MG 28 TAB | Brexpiprazole | Antipsychotics |
| REXULTI 2 MG 28 TAB | Brexpiprazole | Antipsychotics |
| REXULTI 3 MG 28 TAB | Brexpiprazole | Antipsychotics |
| REXULTI 4 MG 28 TAB | Brexpiprazole | Antipsychotics |
| RISPOND 1 MG 20 TAB | Risperidone | Antipsychotics |
| RISPOND 2 MG 20 TAB | Risperidone | Antipsychotics |
| RISPOND 3 MG 20 TAB | Risperidone | Antipsychotics |
| RISPOND 4 MG 20 TAB | Risperidone | Antipsychotics |
| RISPERIDEX 1 MG 20 TAB | Risperidone | Antipsychotics |
| RISPERIDEX 2 MG 20 TAB | Risperidone | Antipsychotics |
| RISPERIDEX 3 MG 20 TAB | Risperidone | Antipsychotics |
| RISPERIDEX 4 MG 20 TAB | Risperidone | Antipsychotics |
| QUETIAPINE INV SR 50MG 30 | Quetiapine | Antipsychotics |
| QUETIAPINE INV SR150MG 30 | Quetiapine | Antipsychotics |
| QUETIAPINE INV SR200MG 30 | Quetiapine | Antipsychotics |
| QUETIAPINE INV SR300MG 30 | Quetiapine | Antipsychotics |
| QUETIAPINE INV SR400MG 30 | Quetiapine | Antipsychotics |
| SEROQUEL 300 MG 30 TAB | Quetiapine | Antipsychotics |
| TREVICTA PFS 175 MG SYRNG | Paliperidone | Antipsychotics |
| TREVICTA PFS 263 MG SYRNG | Paliperidone | Antipsychotics |
| TREVICTA PFS 350 MG SYRNG | Paliperidone | Antipsychotics |
| TREVICTA PFS 525 MG SYRNG | Paliperidone | Antipsychotics |
| NOZINAN 25 MG 20 TAB | Levomepromazine | Antipsychotics |
| RISPOND ORAL SOL 30ML | Risperidone | Antipsychotics |
| SEROQUELXR 50 MG 30 TAB | Quetiapine | Antipsychotics |
| SEROQUELXR 200 MG 30 TAB | Quetiapine | Antipsychotics |
| SEROQUELXR 300 MG 30 TAB | Quetiapine | Antipsychotics |
| SEROQUELXR 400 MG 30 TAB | Quetiapine | Antipsychotics |
| ABILIFY 5 MG 28 TAB | Aripiprazole | Antipsychotics |
| ABILIFY 10 MG 28 TAB | Aripiprazole | Antipsychotics |
| ABILIFY 15 MG 28 TAB | Aripiprazole | Antipsychotics |
| ABILIFY 30 MG 28 TAB | Aripiprazole | Antipsychotics |
| ABILIFY MAINTENA 400MG SR | Aripiprazole | Antipsychotics |
| SERDOLECT 4 MG 30 TAB | Sertindole | Antipsychotics |
| SERDOLECT 16 MG 28 TAB | Sertindole | Antipsychotics |
| INVEGA 3 MG 28 TAB | Paliperidone | Antipsychotics |
| INVEGA 6 MG 28 TAB | Paliperidone | Antipsychotics |
| RISPERDAL CONS SMART 25MG | Risperidone | Antipsychotics |
| RISPERDAL CONS SMART 37.5 | Risperidone | Antipsychotics |
| RISPERDAL CONS SMART 50MG | Risperidone | Antipsychotics |
| INVEGA 9 MG 28 TAB | Paliperidone | Antipsychotics |
| NEULEPTIL 4% DROPS | Periciazine | Antipsychotics |
| NOZINAN 100 MG 20 TAB | Levomepromazine | Antipsychotics |
| FLUPHENAZINE 25MG_1ML 29G | Fluphenazine | Antipsychotics |
| MODITEN DEP 25MG_1ML 29-G | Fluphenazine | Antipsychotics |
| NEULEPTIL 4% DROPS 29-G | Periciazine | Antipsychotics |
| NEULEPTIL 4% DROPS 29-G | Periciazine | Antipsychotics |
| DEPIXOL 20MG/ML 1ML 29-G | Flupentixol | Antipsychotics |
| PERICATE 1 ML* 5 AMP | Haloperidol | Antipsychotics |
| HALDOL 5 AMP | Haloperidol | Antipsychotics |
| ORAP 4 MG 20 TAB 29-G | Pimozide | Antipsychotics |
| ARIPIPRAZOL 1MG/1ML100ML | Aripiprazole | Antipsychotics |
| TAROCTYL 25MG/5ML I.M | Chlorpromazine | Antipsychotics |
| TAROCTYL 50 MG 2 ML 5 | Chlorpromazine | Antipsychotics |
| MODITEN D 25MG/ML 1A 29-G | Fluphenazine | Antipsychotics |
| DEPIXOL 3MG 100TAB 29-G | Flupentixol | Antipsychotics |
| ETUMINA 40 MG 30 TAB 29-G | Clotiapine | Antipsychotics |
| PERIDOR 10 MG 60 TAB | Haloperidol | Antipsychotics |
| LOZAPINE 25 MG 50 TAB | Clozapine | Antipsychotics |
| LEPONEX 25 MG 50 TAB | Clozapine | Antipsychotics |
| LEPONEX 100 MG 50 TAB | Clozapine | Antipsychotics |
| LOZAPINE 100 MG 50 TA | Clozapine | Antipsychotics |
| METHOZANE 25 MG 1000 TAB | Levomepromazine | Antipsychotics |
| METHOZANE 25 MG 30 TAB | Levomepromazine | Antipsychotics |
| METHOZANE 100 MG 30 TAB | Levomepromazine | Antipsychotics |
| METHOZANE 100 MG 1000 TAB | Levomepromazine | Antipsychotics |
| LARGACTIL 100 MG 20T 29-G | Chlorpromazine | Antipsychotics |
| LARGACTIL 25 MG 50 T 29-G | Chlorpromazine | Antipsychotics |
| LARGACTIL 100 MG 30T 29-G | Chlorpromazine | Antipsychotics |
| SOLIDON 100 MG 50TAB 29-G | Chlorpromazine | Antipsychotics |
| CHLORPROMAZINE 100MG 29-G | Chlorpromazine | Antipsychotics |
| LARGACTIL 25 MG 60 T 29-G | Chlorpromazine | Antipsychotics |
| LARGACTIL 100 MG 60T 29-G | Chlorpromazine | Antipsychotics |
| LARGACTIL 25 MG 25 T 29-G | Chlorpromazine | Antipsychotics |
| CHLORPROMAZINE 100MG 29-G | Chlorpromazine | Antipsychotics |
| ACEMAP 20 MG 10 TAB 29-G | Penfluridol | Antipsychotics |
| SEMAP 20 MG 12 TAB 29-G | Penfluridol | Antipsychotics |
| SEREPRILE 100 MG 20T 29-G | Tiapride | Antipsychotics |
| TIAPRIZAL 100 MG 24T 29-G | Tiapride | Antipsychotics |
| TIAPRIDE 100 MG 20 T 29-G | Tiapride | Antipsychotics |
| CLOTIAPINE 20MG 120 CAP | Clotiapine | Antipsychotics |
| THIORIDAZIN 25MG 100 29-G | Thioridazine | Antipsychotics |
| THIORIDAZIN 100MG 100 29G | Thioridazine | Antipsychotics |
| THIORIDAZIN 10MG 100 29-G | Thioridazine | Antipsychotics |
| CHLORPROMAZIN 25MG 50 29G | Chlorpromazine | Antipsychotics |
| HALDOL DECN 100MG_ML5 29G | Haloperidol | Antipsychotics |
| THIORIDAZIN 25MG 100 29-G | Thioridazine | Antipsychotics |
| FLUPHENAZIN 25MG/ML 5 29G | Fluphenazine | Antipsychotics |
| HALOPERIDOL 2MG/ML DR 29G | Haloperidol | Antipsychotics |
| CHLOPROMAZINE 100MG 50 29 | Chlorpromazine | Antipsychotics |
| NEULEPTIL 10MG 30TAB 29-G | Periciazine | Antipsychotics |
| OLANZAPINE 10 MG 28 TAB F | Olanzapine | Antipsychotics |
| OLANZAPINE 5 MG 28 TAB F | Olanzapine | Antipsychotics |
| OLANZAPINE 7.5 MG 56TAB F | Olanzapine | Antipsychotics |
| ASSIVAL 10MG/2ML 5 AMP | Diazepam | Anxiolytics |
| FRISIUM 10 MG 20 TAB | Clobazam | Anxiolytics |
| ALTMAN PROBI VAGINAL | Clobazam | Anxiolytics |
| GLORIUM 5 MG 20 CAP | Medazepam | Anxiolytics |
| GLORIUM 10 MG 20 CAP | Medazepam | Anxiolytics |
| LENITIN 1.5 MG 20 TAB | Bromazepam | Anxiolytics |
| LENITIN 3 MG 20 TAB | Bromazepam | Anxiolytics |
| LENITIN 6 MG 10 TAB | Bromazepam | Anxiolytics |
| MEPRO 400 MG 20 TAB | Meprobamate | Anxiolytics |
| OTAREX 10 MG 20 TAB O | Hydroxyzine | Anxiolytics |
| OTAREX 25 MG 20 TAB | Hydroxyzine | Anxiolytics |
| SERVIUM 10 MG 20 DRAG | Chlordiazepoxide | Anxiolytics |
| SERVIUM 5 MG 30T | Chlordiazepoxide | Anxiolytics |
| VABEN FORTE 30 MG 30 | Oxazepam | Anxiolytics |
| VABEN 10 MG 30 TAB | Oxazepam | Anxiolytics |
| TRANXAL 15 MG 30 TAB | Potassium Clorazepate | Anxiolytics |
| TRANXAL 5 MG 30 TAB | Potassium Clorazepate | Anxiolytics |
| LORIVAN 1 MG 50 TAB | Lorazepam | Anxiolytics |
| DISOPAM 5 MG 30 TAB | Diazepam | Anxiolytics |
| DISOPAM 10 MG 30 TAB | Diazepam | Anxiolytics |
| XANAGIS 0.25 MG 50 TA | Alprazolam | Anxiolytics |
| XANAGIS 0.5 MG 50 TAB | Alprazolam | Anxiolytics |
| XANAGIS 1 MG 50 TAB | Alprazolam | Anxiolytics |
| STESOLID 5 MG ENEMA 2 TUB | Diazepam | Anxiolytics |
| STESOLID 10 MG ENEMA 2TUB | Diazepam | Anxiolytics |
| BUSPIROL 10 MG 60 TAB | Buspirone | Anxiolytics |
| SORBON 5 MG 30 TAB | Buspirone | Anxiolytics |
| SORBON 10 MG 30 TAB | Buspirone | Anxiolytics |
| ALPRALID 0.25 MG 30 TAB | Alprazolam | Anxiolytics |
| ALPRALID 0.5 MG 30 TAB | Alprazolam | Anxiolytics |
| ALPRALID 1 MG 30 TAB | Alprazolam | Anxiolytics |
| ALPROX 1 MG 30 TAB | Alprazolam | Anxiolytics |
| ALPROX 0.5 MG 30 TAB | Alprazolam | Anxiolytics |
| ALPROX 0.25 MG 30 TAB | Alprazolam | Anxiolytics |
| STESOLID 5 MG ENEMA 5 TUB | Diazepam | Anxiolytics |
| STESOLID 10 MG ENEMA 5 TU | Diazepam | Anxiolytics |
| DIAZEPAM-RAT 10MG/2ML 29G | Diazepam | Anxiolytics |
| ASSIVAL 5 MG 30 TAB | Diazepam | Anxiolytics |
| ASSIVAL 10 MG 30 TAB | Diazepam | Anxiolytics |
| ASSIVAL 2 MG 30 TAB | Diazepam | Anxiolytics |
| OTAREX INF. 12 SUPP | Hydroxyzine | Anxiolytics |
| LORIVAN 1 MG 20 TAB | Lorazepam | Anxiolytics |
| SORBON 15 MG 30 TAB | Buspirone | Anxiolytics |
| ASSIVAL TEVA 10MG/2ML 10A | Diazepam | Anxiolytics |
| FRISIUM 10 MG 30 TAB | Clobazam | Anxiolytics |
| OTAREX S 2MG_ML 100 PREP | Hydroxyzine | Anxiolytics |
| DIAZEPAM 10MG/2ML AM 29-G | Diazepam | Anxiolytics |
| DIAZEPAM RECTAL 5 MG 29-G | Diazepam | Anxiolytics |
| DIAZEPAM RECTAL 10MG 29-G | Diazepam | Anxiolytics |
| DIAZEPAM RECTAL 10MG 29-G | Diazepam | Anxiolytics |
| DIAZEPAM RECTAL 5 MG 29-G | Diazepam | Anxiolytics |
| STESOLID RECTAL 5 MG 29-G | Diazepam | Anxiolytics |
| STESOLID RECTAL 10MG 29-G | Diazepam | Anxiolytics |
| DIAZEPAM 5MG 60T 29-G RAZ | Diazepam | Anxiolytics |
| DIAZEPAM 10MG 60T 29G RAZ | Diazepam | Anxiolytics |
| DIAZEPAM 10MG 28T 29G MED | Diazepam | Anxiolytics |
| DIAZEPAM 5MG 28T 29-G MED | Diazepam | Anxiolytics |
| HYDROXYZINE10MG/1ML100ML | Hydroxyzine | Anxiolytics |
| DIAZEPEKS 5MG 20T 29G MED | Diazepam | Anxiolytics |
| DIAZEPAM 5MG RECTL 5 29-G | Diazepam | Anxiolytics |
| DIAZEPAM DESIT R 10MG 29G | Diazepam | Anxiolytics |
| CALMANERVIN 20 TAB | Hypnotics and Sedatives In Combination, Excl. Barbiturates | Hypnotics and Sedatives |
| DORMICUM 5 MG/1 ML 10 AM | Midazolam | Hypnotics and Sedatives |
| NERVEN DRAGEES 20 TAB | Valerian Combinations | Hypnotics and Sedatives |
| HYPNODORM 10 TAB | Flunitrazepam | Hypnotics and Sedatives |
| NUMBON 20 TAB | Nitrazepam | Hypnotics and Sedatives |
| ROHYPNOL 10 TAB | Flunitrazepam | Hypnotics and Sedatives |
| TRICLONAM SYR | Triclofos | Hypnotics and Sedatives |
| SONGHA NIGHT 30 TAB | Valerian | Hypnotics and Sedatives |
| ZODORM 10 MG 20 TAB | Zolpidem | Hypnotics and Sedatives |
| VALERIAN DROPS 10 ML FL | Valerian | Hypnotics and Sedatives |
| VALERIAN DROPS 20 ML FL | Valerian | Hypnotics and Sedatives |
| CALMANERVIN S ELIXIR | Hypnotics and Sedatives In Combination, Excl. Barbiturates | Hypnotics and Sedatives |
| BONDORMIN 0.25 MG 10 TAB | Brotizolam | Hypnotics and Sedatives |
| VALETON | Valerian | Hypnotics and Sedatives |
| VALERIAN TINCTURE SAM | Valerian | Hypnotics and Sedatives |
| VALERIAN 50 ML BDZ MARSHL | Valerian | Hypnotics and Sedatives |
| DORMICUM 5 MG/5ML 10 AMP | Midazolam | Hypnotics and Sedatives |
| PASSIFLORA COMP SYR VIT | Other Hypnotics and Sedatives | Hypnotics and Sedatives |
| VALERIAN DROPS 10 ML VIT | Valerian | Hypnotics and Sedatives |
| STILNOX 10 MG 20 TAB OLD | Zolpidem | Hypnotics and Sedatives |
| MELATONIN1MG/1ML100ML | Melatonin | Hypnotics and Sedatives |
| CALMANERVINE DAY 30 TAB | Hypnotics and Sedatives In Combination, Excl. Barbiturates | Hypnotics and Sedatives |
| CALMANERVIN NIGHT 20 TAB | Hypnotics and Sedatives In Combination, Excl. Barbiturates | Hypnotics and Sedatives |
| BROTIZOLAM TEVA 0.25MG 10 | Brotizolam | Hypnotics and Sedatives |
| CIRCADIN 2 MG 30 TAB | Melatonin | Hypnotics and Sedatives |
| NOCTURNO LS 3.75 MG 20 T | Zopiclone | Hypnotics and Sedatives |
| NOCTURNO FORTE 7.5 20 TAB | Zopiclone | Hypnotics and Sedatives |
| MIDOLAM 1 MG/ML 5ML 5 A | Midazolam | Hypnotics and Sedatives |
| MIDOLAM 5 MG/ML 5 AMP | Midazolam | Hypnotics and Sedatives |
| BUCCOLAM 10MG 4X2 ML | Midazolam | Hypnotics and Sedatives |
| BUCCOLAM 2.5MG 4X0.5ML | Midazolam | Hypnotics and Sedatives |
| BUCCOLAM 5 MG 4X1 ML | Midazolam | Hypnotics and Sedatives |
| BUCCOLAM 7.5MG 4X1.5ML | Midazolam | Hypnotics and Sedatives |
| VALERIAN TINCTURE | Valerian | Hypnotics and Sedatives |
| MELATONIN CAP 3 MG PREP | Melatonin | Hypnotics and Sedatives |
| MELATONIN CAP 4 MG PREP | Melatonin | Hypnotics and Sedatives |
| MELATONIN CAP 5 MG PREP | Melatonin | Hypnotics and Sedatives |
| MELATONIN SYR PREP | Melatonin | Hypnotics and Sedatives |
| IMOVANE 7.5 MG 20 TAB | Zopiclone | Hypnotics and Sedatives |
| ZOPICLONE 7.5 MG 20 TAB | Zopiclone | Hypnotics and Sedatives |
| RELAXINE 15 TAB | Valerian | Hypnotics and Sedatives |
| RELAXINE 15 TAB GSL | Valerian | Hypnotics and Sedatives |
| SLENYTO 1 MG 60 PR TAB | Melatonin | Hypnotics and Sedatives |
| SLENYTO 5 MG 30 PR TAB | Melatonin | Hypnotics and Sedatives |
| CIRCADIN 2 MG 21 TAB | Melatonin | Hypnotics and Sedatives |
| STILNOX 10 MG 20 TAB | Zolpidem | Hypnotics and Sedatives |
| AMBIEN CR 6.25 MG 28 TAB | Zolpidem | Hypnotics and Sedatives |
| AMBIEN CR 12.5 MG 28 TAB | Zolpidem | Hypnotics and Sedatives |
| CALMANERVIN 30 TAB | Hypnotics and Sedatives In Combination, Excl. Barbiturates | Hypnotics and Sedatives |
| CALMANERVIN 10 TAB | Hypnotics and Sedatives In Combination, Excl. Barbiturates | Hypnotics and Sedatives |
|  |  |  |
|  |  |  |

Supplementary Table 5. ICD 9 codes for Bacterial Pneumonia

| ICD-9 | Name |  |
| --- | --- | --- |
| 481 | PNEUMOCOCCAL PNEUMONIA [STREPTOCOCCUS PNEUMONIAE PNEUMONIA] | Bacterial Pneumonia |
| 482 | PNEUMONIA, BACTERIAL, OTHER | Bacterial Pneumonia |
| 482.0 | PNEUMONIA DUE TO KLEBSIELLA PNEUMONIAE | Bacterial Pneumonia |
| 482.1 | PNEUMONIA DUE TO PSEUDOMONAS | Bacterial Pneumonia |
| 482.2 | PNEUMONIA DUE TO HEMOPHILUS INFLUENZAE (H. INFLUENZAE) | Bacterial Pneumonia |
| 482.3 | PNEUMONIA DUE TO STREPTOCOCCUS | Bacterial Pneumonia |
| 482.30 | PNEUMONIA DUE TO STREPTOCOCCUS, UNSPECIFIED | Bacterial Pneumonia |
| 482.31 | PNEUMONIA DUE TO STREPTOCOCCUS, GROUP A | Bacterial Pneumonia |
| 482.32 | PNEUMONIA DUE TO STREPTOCOCCUS, GROUP B | Bacterial Pneumonia |
| 482.39 | PNEUMONIA DUE TO OTHER STREPTOCOCCUS | Bacterial Pneumonia |
| 482.4 | PNEUMONIA DUE TO STAPHYLOCOCCUS | Bacterial Pneumonia |
| 482.40 | PNEUMONIA DUE TO STAPHYLOCOCCUS, UNSPECIFIED | Bacterial Pneumonia |
| 482.41 | METHICILLIN SUSCEPTIBLE PNEUMONIA DUE TO STAPHYLOCOCCUS AUREUS | Bacterial Pneumonia |
| 482.42 | METHICILLIN RESISTANT PNEUMONIA DUE TO STAPHYLOCOCCUS AUREUS | Bacterial Pneumonia |
| 482.8 | PNEUMONIA, BACTERIAL, OTHER SPECIFIED BACTERIA | Bacterial Pneumonia |
| 482.81 | PNEUMONIA DUE TO ANAEROBES | Bacterial Pneumonia |
| 482.82 | PNEUMONIA DUE TO ESCHERICHIA COLI [E.COLI] | Bacterial Pneumonia |
| 482.83 | PNEUMONIA DUE TO OTHER GRAM-NEGATIVE BACTERIA | Bacterial Pneumonia |
| 482.84 | LEGIONNAIRE'S DISEASE | Bacterial Pneumonia |
| 482.89 | PNEUMONIA DUE TO OTHER SPECIFIED BACTERIA | Bacterial Pneumonia |
| 482.9 | PNEUMONIA, BACTERIAL, UNSPECIFIED | Bacterial Pneumonia |

Supplementary Table 6. ICD 9 codes for Organ-Specific Autoimmune Disorders

| ICD-9 | Name |  |
| --- | --- | --- |
| 242.011 | GRAVES' DISEASE-HYPERTHYROIDISM | Organ Specific |
| 245.20 | HASHIMOTO'S DISEASE | Organ Specific |
| 252.10 | PARATHYROIDITIS (AUTOIMMUNE) | Organ Specific |
| 255.41 | ADDISON'S MELANODERMA (ADRENAL CORTICAL HYPOFUNCTION) | Organ Specific |
| 255.40 | ADRENAL ATROPHY (AUTOIMMUNE) | Organ Specific |
| 581.11 | IDIOPATHIC MEMBRANOUS GLOMERULAR DISEASE | Organ Specific |
| 340 | MULTIPLE SCLEROSIS | Organ Specific |
| 341 | Other demyelinating disorders | Organ Specific |
| 357.01 | GUILLAIN BARRE DISEASE OR SYNDROME | Organ Specific |
| 358 | MYASTHENIA GRAVIS | Organ Specific |
| 694.0 | Dermatitis herpetiformis | Organ Specific |
| 704.0 | ALOPECIA | Organ Specific |
| 708.1 | URTICARIA | Organ Specific |
| 250.01 | TYPE 1 DIABETES | Organ Specific |
| 255.41 | GLUCOCORTICOID DEFICIENCY (PRIMARY ADRENAL INSUFFICIENCY)-ADDISON'S DISEASE (BRONZE) | Organ Specific |
| 283.0 | AUTOIMMUNE HEMOLYTIC ANEMIAS | Organ Specific |
| 283.00 | AUTOIMMUNE HEMOLYTIC DISEASE (COLD TYPE) (WARM TYPE) | Organ Specific |
| 283.0 | AUTOIMMUNE HEMOLYTIC ANEMIAS | Organ Specific |
| 287 | PURPURA AND OTHER HEMORRHAGIC CONDITIONS | Organ Specific |
| 245.22 | THYROIDITIS, AUTOIMMUNE | Organ Specific |
| 393 | PERICARDITIS, RHEUMATIC, CHRONIC | Organ Specific |
| 571.42 | CHRONIC AUTOIMMUNE HEPATITIS | Organ Specific |
| 555 | CROHN'S DISEASE - REGIONAL ENTERITIS | Organ Specific |
| 556 | ULCERATIVE ENTEROCOLITIS | Organ Specific |
| 696 | PSORASIS | Organ Specific |
| 709.01 | Vitiligo | Organ Specific |
| 714 | RHEUMATOID ARTHRITIS AND OTHER INFLAMMATORY POLYARTHROPATHIES | Organ Specific |

Supplementary Table 7: List of laboratory tests examined in study


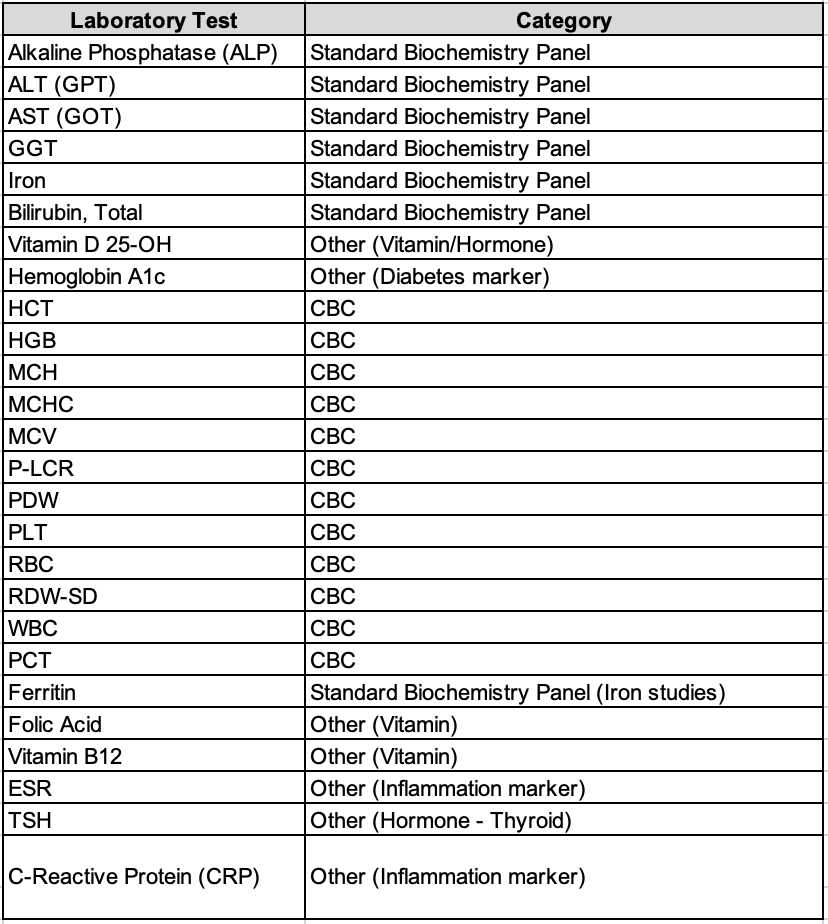

Supplement: Supplementary file 1 — Supplementary Tables [file 41380_2026_3515_MOESM1_ESM.docx]
