# Supplementary material for: Intensity and exposure proximity as determinants of differential stress-related health outcomes
Source: Mol Psychiatry. 2026 Mar 6;31(7):3955–64. doi: 10.1038/s41380-026-03515-5 (PMC13269130; doi:10.1038/s41380-026-03515-5)
Supplement: Supplementary file 2 — Supplementary Text [file 41380_2026_3515_MOESM2_ESM.docx]

Supplementary Text

Antipsychotic use increased in young men in several shelter times of all exposures: (p<0.001, 15s pre vs. 15s post; p<0.05, 30s pre vs 30s post; p<0.05, 90s pre vs 90s post, (Supplementary Figure 1A, bottom, left inset) with the most dramatic increase in high exposure areas at 3%, in women 19-45 (Figure Supplementary 1A upper, left inset) at 2.5% in high exposed regions (p<0.001, 15s pre vs 15s post). However, unlike other drug classes such as antidepressants and anxiolytics, in which in the high exposure areas, women have higher consumption, antipsychotic consumption is not significant between sexes. Rates of acute psychosis (ICD10: F23) and psychosis spectrum were examined but were very small in the 15s group. In other exposure 30s and 90sgroups, the prevalence mirrored the medication use at about 1-2% of the population, but we could not make an exposure-based comparison as we did with other diagnoses and medications.

Increases in hypnotics and sedatives (Supplementary Figure 1B) were seen in young men of high and moderate exposure (p<0.001, 15s pre vs. 15s post, p<0.05, 30s pre vs. 30s post). In women of high exposure areas in the two youngest age groups there was an increase in usage of 6% (p<0.0001 19-45, 15s pre vs post; p<0.001, 46-64 15s pre vs 15s post) with prevalence up to 10% post October 7th. Uniquely, hypnotics and sedatives usage were highest in 2015-2019 already in women 65+, especially those in high exposure areas with use of up to 30%.

Women in general, also had higher prevalence rates than men (Supplementary Figure 2) overall post-Oct 7th in all shelter times (p<0.001, 15s F vs 15s M; p<0.001 30s F vs 30s M; p<0.001; 45s F vs 45s M; p<0.001 60s F vs 60s M; 90s F vs 90s M).

Using logistic regression, examining factors of shelter time, timing, and sex, we demonstrate that the 15s shelter time had the biggest increase from baseline in % prevalence of psychiatric load in all age groups post October 7th (p<0.0001 19-45, 46-64, 65+ 15s M, 15s F,). In moderately exposed areas, in contrast, the most affected groups post October 7th were 30s and 45s, specifically younger women (p<0.0001, 30s 19-45 F; p<0.0001 45s 19-45F, p<0.001 30s, 46-64 F, p<0.05 45s 46-64 F) followed by younger moderately exposed men (p<0.001 45s 19-45 M, p<0.05 45s 46-64 M).

WBC levels (Figure 3A) in women 19-45 were also significant in several years, but with very small effect size (15vs 90s, 19-45, 2005, 2009, 2011-2016, p<0.01).In contrast MCV levels were elevated in moderately exposed women and men chronically elevated in all years in both 19-45 and 46-64 age with very small effect size of under 0.20, (women, 30s vs 90s, 19-45 2005-2024 p<0.0; 46-64, 2005-2024 p<0.01; men, 30s vs 90s.

Figure Legend

Supplementary Figure 1. Antipsychotic and Sedative Use January 2005- August 2024

Antipsychotic use increased in young men in several shelter times of all exposures: (p<0.001, 15s pre vs. 15s post; p<0.05, 30s pre vs 30s post; p<0.05, 90s pre vs 90s post, (Supplementary Figure 1B, left inset) with the most dramatic increase in high exposure areas at 3%, in women 19-45 (Figure Supplementary 1A left inset) at 2.5% in high exposed regions (p<0.001, 15s pre vs 15s post). However, unlike other drug classes such as antidepressants and anxiolytics, in which in the high exposure areas, women have higher consumption, antipsychotic consumption is not significant between sexes .

Increases in hypnotics and sedatives (Figure Supplementary 1B) were seen in young men of high and moderate exposure (p<0.001, 15s pre vs. 15s post, p<0.05, 30s pre vs. 30s post). In women of high exposure areas in the two youngest age groups there was an increase in usage of 6% (p<0.001 19-45, 15s pre vs post; p<0.001, 46-64 15s pre vs 15s post) with prevalence up to 10% post October 7th. Uniquely, hypnotics and sedatives usage were highest in 2015-2019 already in women 65+, especially those in high exposure areas with use of up to 30%.
