# Supplementary figures and images for: Intensity and exposure proximity as determinants of differential stress-related health outcomes
Source: Mol Psychiatry. 2026 Mar 6;31(7):3955–64. doi: 10.1038/s41380-026-03515-5 (PMC13269130; doi:10.1038/s41380-026-03515-5)

A

## Antipsychotics

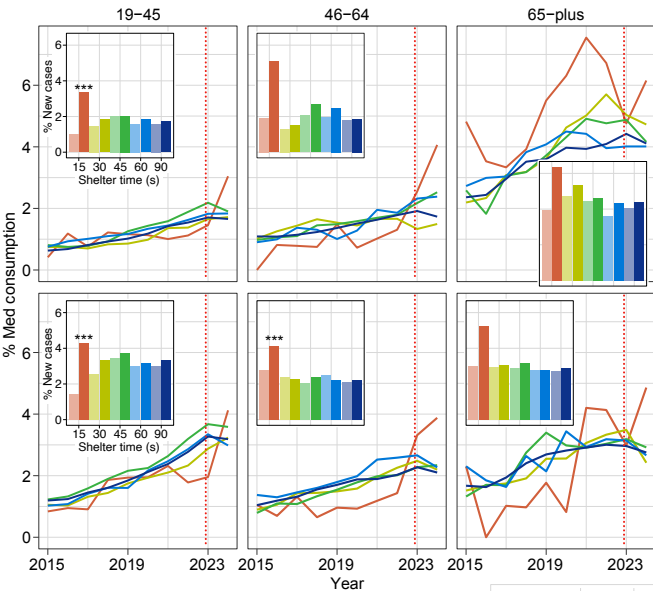

B

## Sedatives &amp; Hypnotics

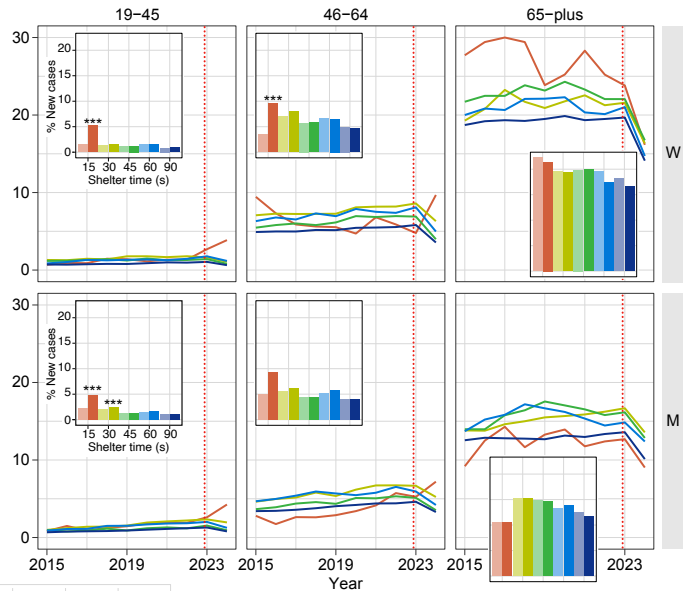

Supplement: Supplementary file 3 — Supplementary Figure 1 [file 41380_2026_3515_MOESM3_ESM.pdf]
